# Management of ovarian granulosa cell tumor in childhood: a case report and recommendations for a multidisciplinary approach

**DOI:** 10.3389/fonc.2025.1634166

**Published:** 2025-11-24

**Authors:** Sofia Maria Carlotta Arnaboldi, Giovanna Gattuso, Maria Laura Nicolosi, Silvia Molinari, Laura Rachele Bettini, Ernesto Leva, Paolo Passoni, Valentina Chiappa, Cecilia Daolio, Adriana Cristina Balduzzi, Maura Massimino, Monica Terenziani, Alessandro Cattoni

**Affiliations:** 1 Department of Pediatrics, Fondazione IRCCS San Gerardo dei Tintori, Monza, Italy; 2 Pediatric Unit, Fondazione IRCCS Istituto Nazionale dei Tumori, Milan, Italy; 3 Department of Medicine and Surgery, Università degli studi di Milano-Bicocca, Milan, Italy; 4 Department of Medicine and Surgery, Università degli studi di Milano, Milan, Italy; 5 Fondazione IRCCS Ca’ Granda, Ospedale Maggiore Policlinico di Milano, Milan, Italy; 6 Department of Gynecology and Obstetrics, Fondazione IRCCS San Gerardo dei Tintori, Monza, Italy; 7 Gynecology Oncology, IRCCS Istituto Nazionale dei Tumori, Milan, Italy

**Keywords:** sex-cord stromal tumor, granulosa cell tumor, rare tumors management, ovarian cancers, pediatric gynecologic cancers, cancer predisposing syndromes, precocious puberty, cancer survivorship care plan

## Abstract

Juvenile granulosa cell tumor (jGCT) is a rare subtype of pure sex-cord tumors, mostly affecting patients under 20 years of age. As this tumor originates from ovarian cells specialized in sex steroid secretion, jGCT can present with endocrine disorders, including precocious puberty in prepubertal girls or menstrual irregularities in postpubertal patients. In other cases, jGCT may manifest with symptoms related to pelvic mass effects or acute abdomen, prompting urgent gynecologic or surgical evaluation. Most patients are diagnosed with ovarian-confined disease, and for these patients, the survival rates exceed 90% following surgery alone. However, advanced and relapsed disease remains a significant concern. As the survival rates for cancer continue to improve, addressing survivorship care is essential. Long-term follow-up for patients diagnosed with jGCT in childhood requires a multidisciplinary approach. We hereby describe a clinical case of jGCT diagnosed in an infant girl for whom a comprehensive multidisciplinary care plan was arranged. Through a comprehensive review of the literature, we developed a clinically applicable flowchart for the multidisciplinary management of jGCT at diagnosis and during follow-up, emphasizing the need for patient-centered care that integrates the work of oncologists, endocrinologists, surgeons, gynecologists, and geneticists.

## Introduction

Ovarian sex-cord stromal tumors (OSCSTs) are neoplasms originating from two cellular components of the ovary: peri-ovocytic follicular cells (also referred to as sex cord cells, including granulosa and Sertoli cells) and stromal cells (including fibroblasts, theca cells, and Leydig cells). These tumors are classified as *pure sex-cord tumors*, *pure stromal tumors*, or *mixed sex-cord stromal tumors* based on the presence of one or more cancerous cell types.

Within the subset of sex-cord tumors, granulosa cell tumors (GCTs) consist of primitive granulosa cells arranged in solid and follicular patterns, which can exhibit a wide spectrum of malignancy potential. GCTs are further classified into adult and juvenile types based on clinical and histopathological features (1–3). However, immunohistochemical markers and genetic testing on histological specimens are often necessary for definitive differentiation between the two subtypes (4, 5).

Although overall rare in childhood, with an incidence of as low as two per 1,000,000 annually, jGCTs approximately represent 15% of all pediatric ovarian neoplasms and 70% of sex-cord stromal tumors occurring in patients aged less than 20 years (6, 7).

Since these tumors originate from ovarian cells specialized in sex steroid secretion, jGCT can present with clinical and biochemical signs consistent with isosexual or heterosexual peripheral precocious puberty in prepubertal girls or with menstrual irregularities and secondary amenorrhea in postpubertal girls (8). In contrast, non-secreting tumors often present symptoms related to pelvic mass effects, ranging from recurrent abdominal pain, pollakiuria, and sub-occlusive symptoms to acute abdomen caused by ovarian torsion or tumor rupture. These scenarios typically prompt urgent evaluation and surgical intervention by gynecologists or pediatric surgeons.

Due to their rarity, most of the available data on the clinical presentation and biological behavior of jGCT are drawn from small case series and retrospective studies. From an oncologic perspective, patients with disease confined to the ovary show an excellent prognosis, with survival rates exceeding 90% following surgery alone (9), and fertility-sparing approach with unilateral salpingo-oophorectomy and/or the preservation of the uterus and partial adnexa are the treatments of choice (10, 11). However, late recurrences or advanced-stage disease are associated with poorer outcomes and require chemotherapy (1). Given the rare occurrence of jGCT, no prospectively generated treatment guidelines are available. Chemotherapy regimens including combinations of cisplatin, etoposide, bleomycin, or ifosfamide (PEB/PEI) and vincristine/cyclophosphamide are used, with additional treatments tailored on a case-by-case basis (5).

While improving survival remains a primary goal, addressing long-term survivorship is equally critical, especially in pediatric oncology. Along with oncologic and gynecologic follow-up, a multidisciplinary approach is warranted for the management of jGCT in childhood. Endocrinological monitoring is crucial over the first months following diagnosis and surgery to assess the regression of estrogenization/virilization, evaluate growth patterns, and detect potential signs consistent with the onset of central precocious puberty, which can arise secondary to hypothalamic priming from tumor-secreted sex steroids (12, 13). In addition, genetic evaluation is also pivotal to identify underlying cancer-predisposing syndromes or other conditions—such as Maffucci syndrome, Ollier disease, disorders of sex development, or other genetic disorders (14–17).

We hereby describe a paradigmatic clinical case of jGCT diagnosed in an infant girl for whom a comprehensive multidisciplinary care plan was arranged.

With the aim of promoting the integrated management of jGCT in childhood, we developed a flowchart that highlights the need for a multidisciplinary approach and clarifies the roles of and challenges for oncologists, endocrinologists, gynecologists, and geneticists involved in the care of affected patients. These recommendations were refined through a comprehensive review of existing guidelines, a systematic analysis of previously published cases of pediatric jGCT, and opinions from experts in pediatric oncology, surgery, endocrinology, and genetics.

## Materials and methods

### Case report

Clinical, laboratory, imaging, and anatomopathological findings are reported, highlighting the pertinent features of this case. Next-generation sequencing (NGS) using a custom panel for overgrowth syndromes (Custom Panel Enrichment and Nextera Flex Enrichment—Illumina) was conducted on peripheral blood with a technical sensitivity of 10%–15%. Sanger sequencing was used for validation. In addition, multiplex ligation-dependent probe amplification (MLPA) for *PTEN* gene was performed to detect *PTEN* copy number variants.

### Systematic review of jGCT case reports

A systematic review was conducted using PubMed to identify previously published case reports and case series on jGCT in childhood. The search was carried out using the following query string in December 2024: ((granulosa cell tumor) OR (sex-cord stromal tumor)) AND ((pediatric)) OR (childhood)). Filter for case reports was applied (*n* = 141). Each article was reviewed for relevance, resulting in a final selection of 46 articles. The following exclusion criteria were applied to the search results: (a) pathology report of non-granulosa cell tumors of the ovary (*n* = 66, including *n* = 7 adult GCT, *n* = 20 sex-cord stromal tumors not otherwise specified or other subtypes, *n* = 39 Sertoli–Leydig cell tumors), (b) non-availability of article full text (*n* = 6, all published between 1960 and 1993), (c) language barriers (*n* = 2 in Danish and *n* = 1 in German), and (d) topic incoherent with the research query (*n* = 18). Clinical cases of pediatric ovarian tumors with mixed histopathological features, but including granulosa cell tumors, were compiled (*n* = 4). Additionally, one article describing extraovarian jGCT was separately described. The references of the included articles were reviewed, and additional relevant articles on ovarian jGCT were integrated. The final list comprised 65 articles, summarized in **Supplementary Tables S4**, **S5**, encompassing a total of 94 cases of jGCT in patients aged <18 years.

## Case presentation

**Figure 1:**
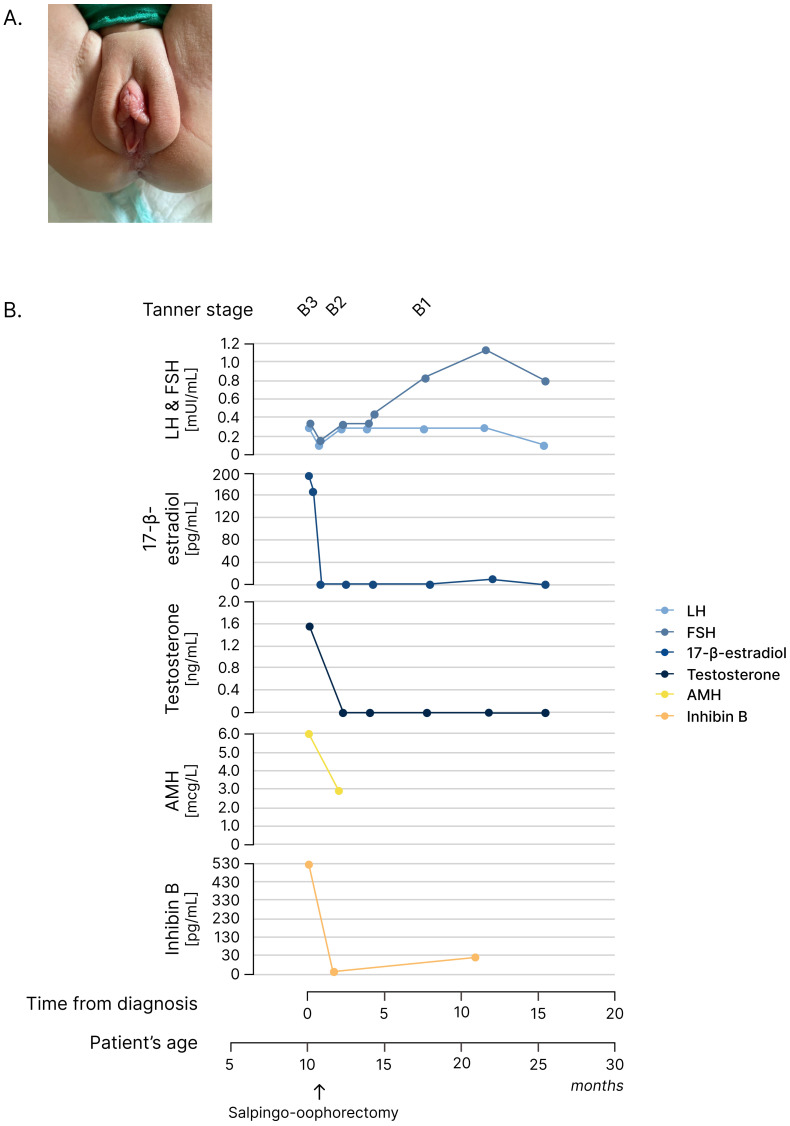
Endocrinologic and oncologic assessment and follow-up. **(A)** Picture of the external genitalia at the initial assessment of the infant girl. **(B)** Line graphs depicting the hypothalamic–pituitary–gonadal axis (LH, FSH, 17-β-estradiol, and testosterone) and tumor markers (AMH and inhibin B) at diagnosis and during follow-up after salpingo-oophorectomy. *AMH*, a*nti-Müllerian* h*ormone; FSH*, f*ollicle-stimulating hormone; LH*, l*uteinizing* h*ormone*.

**Figure 2:**
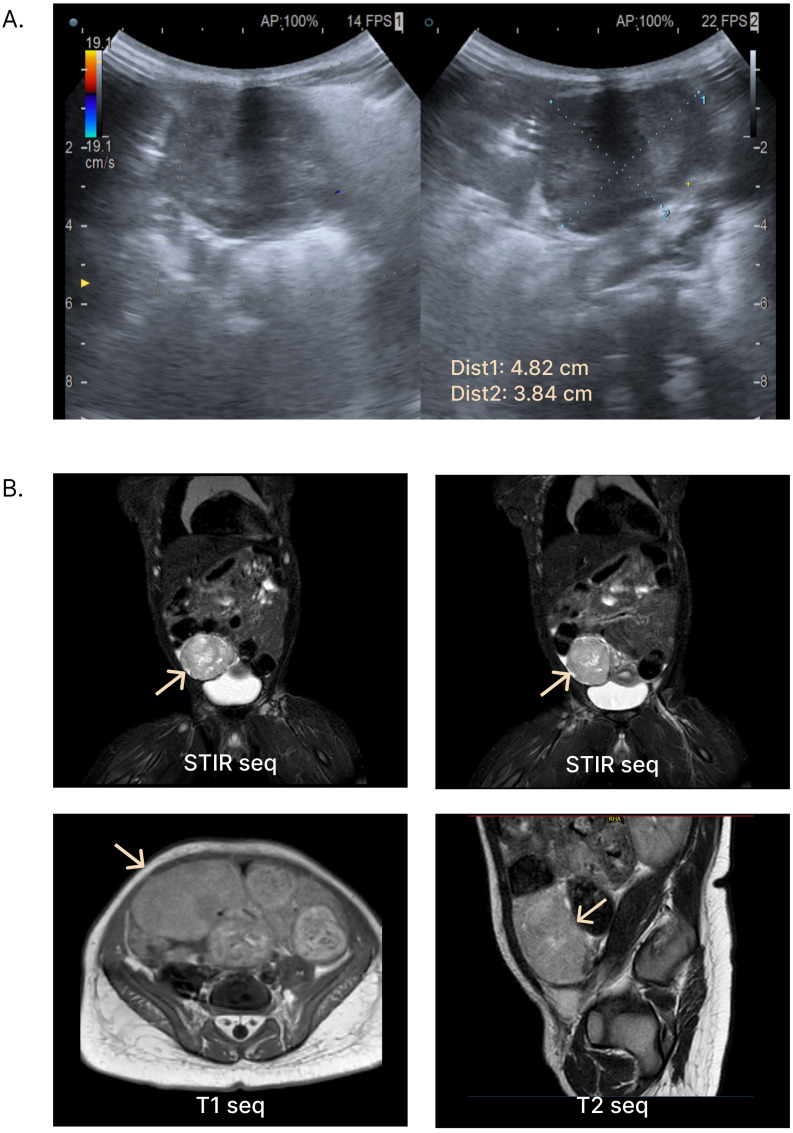
Radiologic diagnostic testing. **(A)** Ultrasound (US) images obtained at diagnosis, revealing a pelvic mass in the right flank region. **(B)** Contrast-enhanced abdominal and pelvic MRI images showing a right pelvic mass originating from the left adnexa, with features suggestive of neoplastic etiology. *Dist*, *distance; Seq*, *sequence*.

A 10-month-old girl was referred to the Pediatric Endocrinology Clinic for specialist evaluation due to bilateral breast enlargement. The patient’s previous medical history was overall unremarkable. She was born at term following an uncomplicated pregnancy. At birth, she was noted to present with relative macrocephaly with a head circumference at the 97th percentile according to WHO charts and a sex-adjusted SDS of 0.83 for weight, 0.53 for length, and 1.91 for head circumference according to National Neonatal Anthropometric Charts (18). However, a neonatal examination and cranial ultrasound showed no pathological findings. Her family history was unremarkable, and neither abnormal pubertal development nor other endocrine disorders were reported. On physical examination, bilateral breast enlargement was observed, without pubarche or axillarche, consistent with Tanner stage B3PH1A1. In addition, the external genitalia appeared estrogenized, with prominent labia minora and majora. Overt clitoromegaly was also noted (**Figure 1A**). No leukorrhea or apocrine sweat smell was present. Macrocephaly with frontal bossing was noted, but growth in weight and length was regular and within the genetic target range. Neurological development was appropriate for her age. The remaining physical examination, including abdominal palpation, was unremarkable with no detectable masses. Given the clinical signs consistent with the co-occurrent secretion of estrogens and androgens (thelarche and estrogenization of genitalia plus clitoromegaly), a picture of peripheral precocious puberty was suspected, and biochemical and hormonal analyses were urgently arranged. As expected, elevated 17-β-estradiol and testosterone in the setting of suppressed gonadotropins (FSH <0.3 mUI/mL, LH <0.3 mUI/mL, 17-β-estradiol 195 pg/mL, and testosterone 1.64 ng/mL) confirmed the clinical suspicion (**Supplementary Table S1**). A 48 × 38-mm mass located in the right pelvic region was detected by performing an urgent abdominal ultrasound (US) (**Figure 2A**). In addition, US outlined radiological findings consistent with remarkable estrogenization of the internal genitalia, with uterine longitudinal diameter exceeding 60 mm, uterine volume of 11 mL, body-to-cervix ratio >1:1, and a stimulated endometrium of 2 mm. Finally, contrast-enhanced pelvic MRI confirmed a solid 56 × 34 × 46-mm lesion located in the right iliac fossa, originating from the contralateral ovary. Neither intraperitoneal fluid nor liver lesions were detected (**Figure 2B**). Along with the clinical, biochemical, and radiologic data gathered, the finding of elevated inhibin B prompted the suspicion of an androgen- and estrogen-secreting granulosa cell tumor of the ovary (**Supplementary Table S2**). Accordingly, the patient underwent a left salpingo-oophorectomy with multiple omental and peritoneal biopsies and peritoneal washing. Pathologic evaluation reported that the ovary was completely replaced by a solid neoplasm with a tense-elastic consistency and a smooth external surface. The microscopic histopathology was consistent with jGCT of the left ovary. The tumor capsule was intact, the fallopian tube had no evident histopathological alterations, and the peritoneum was uninvolved, corresponding to a pathological staging pT1apNx. In addition, a chest X-ray ruled out lung nodules. Overall, final staging corresponded to FIGO/WHO IA. The patient was referred for oncological evaluation. The patient did not receive any adjuvant chemotherapy due to the radical nature of the surgery and the localized stage of the disease at diagnosis. The patient is under a multidisciplinary follow-up involving pediatric oncologists, gynecologists, endocrinologists, and medical geneticists, mirroring the need for comprehensive long-term care. Oncological surveillance includes periodic assessment of inhibin B that was elevated upon diagnosis and normalized following surgical resection (**Figure 1B**; **Supplementary Table S2**), along with three-monthly pelvic US. Endocrine monitoring documented a progressive regression of thelarche and of external genitalia virilization and estrogenization. In addition, the hypothalamic–pituitary–gonadal axis remained quiescent at the last biochemical follow-up (**Figure 1B**). Finally, the co-occurrence of childhood-onset tumor and macrocephaly prompted the referral to our Genetic Clinic in order to rule out underlying overgrowth syndromes. The genetic tests, including a NGS panel for overgrowth syndromes and MLPA testing for *PTEN* copy number variants, showed negative results. Currently, the patient presents no radiologic, clinical, and molecular evidence of disease recurrence 15 months after diagnosis.

## Discussion and literature review

By performing a systematic review of the literature, we identified 94 cases of jGCT in patients diagnosed in childhood or adolescence. The mean age at diagnosis was 7.2 years (range: 0–17 years). When stratified by age, 15 out of 94 patients (15.9%) were infants (<1 year), 42 (44.7%) were aged 1 to 8 years, and 37 (39.4%) were older than 8 years (**Table 1**). Among the 15 infants, two received a prenatal diagnosis of an ovarian mass (19, 20). Clinically significant data at the time of diagnosis and during follow-up are detailed in **Supplementary Tables S4**, **S5** (21–51).

**Table 1:** Demographic and clinical characteristics of the jGCT cases included in the retrospective review.

Characteristics	n° of patients (%)
*Total number of patients included*	*94*
Patients aged ≤ 8 years	57 (60.6%)
• Infants (age < 1 year)	15 (15.9%)
• Age ≥1 and ≤8 years	42 (44.7%)
Post-pubertal patients	27 (28.7%)
*Mean age at diagnosis (range) [years]*	*7.2 (0-17)*

### The point of view of the endocrinologist

Ovarian jGCTs frequently secrete functionally active hormones, leading to endocrine-related clinical manifestations (**Table 2**). By systematically reviewing published literature, we identified precocious thelarche as the most frequently reported presenting sign among patients aged <8 years, with breast budding being the referral reason in 75.4% of the girls aged 8 years or younger. Additional signs of estrogenization in this subgroup included vaginal bleeding (31.6%) and estrogenized external genitalia (17.5%). Moreover, 35.1% of prepubertal girls exhibited signs of heterosexual precocious puberty: pubarche was described in 29.8% of prepubertal girls, clitoromegaly in 8.8%, and axillarche in 5.3%. Remarkably, three cases were diagnosed with jGCT during the neonatal period due to ambiguous genitalia resulting from antenatal testosterone exposure (19, 20, 52).

**Table 2:** Clinical presentation of jGCT in the reviewed cases. The symptoms and signs at presentation in the cases identified from the retrospective review are summarized.

Presenting sign or symptom	n° of patients (%)
Endocrinology	
*Patients aged ≤ 8 years*	*57*
Isosexual precocious puberty	43 (75.4%)
• Vaginal bleeding	18 (31.6%)
• Estrogenized external genitalia	10 (17.5%)
Heterosexual precocious puberty	20 (35.1%)
• Pubarche	17 (29.8%)
• Clitoromegaly	5 (8.8%)
• Axillarche	3 (5.3%)
• Ambigous genitalia	3 (5.3%)
HAIR-AN syndrome	1 (1.7%)
*Post-pubertal patients*	*27*
Menstrual irregularities	11 (40.7%)
Endometrial hyperplasia with bleeding	1 (3.7%)
Gynecology/Pediatric Surgery	
*All patients*	*94*
Any abdominal symptoms	53 (56.4%)
• Abdominal pain	17 (18.1%)
• Abdominal distention	15 (15.9%)
• Anorexia or sub-occlusive symptoms	8 (8.5%)
• Abdominal mass	32 (34.0%)
Meigs syndrome	2 (2.1%)
Ascites	8 (8.5%)
Acute abdomen due to tumor rupture	5 (5.3%)
Uterine torsion	1 (1.1%)
*Post-pubertal patients*	*27*
Any abdominal symptoms	18 (66.7%)
• Abdominal pain or discomfort	10 (37.0%)
• Abdominal distention	7 (25.9%)
• Anorexia or sub-occlusive symptoms	5 (18.5%)
• Abdominal mass	6 (22.2%)
Meigs syndrome	1 (3.7%)
Ascites	5 (18.5%)
Acute abdomen	1 (3.7%)

The number of cases presenting specific symptoms at diagnosis is reported and, when appropriate for diagnostic classification, stratified by patient subgroup (pre-pubertal vs. post-pubertal). HAIR-AN syndrome, hyperandrogenism, insulin resistance, and acanthosis nigricans syndrome.

On the other hand, out of 27 post-pubertal patients, menstrual irregularities or secondary amenorrhea was reported in 40.7% of patients at diagnosis. In one remarkable case, ovarian jGCT was identified following an episode of heavy vaginal bleeding, which required uterine curettage for endometrial hyperplasia (17).

**Figure 3:**
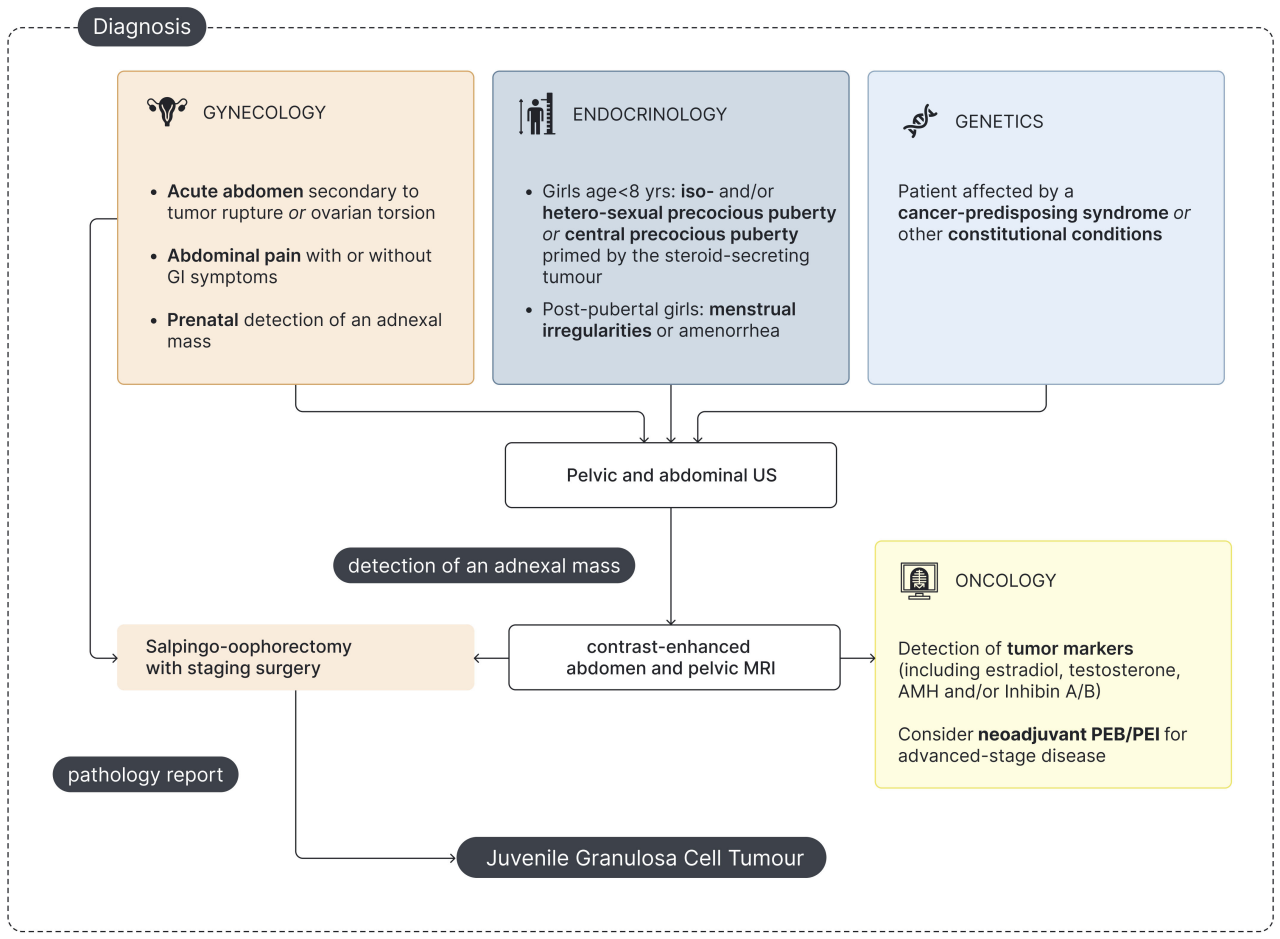
Flowchart for pediatric jGCT management at diagnosis. US - Ultrasound; MRI - Magnetic Resonance Imaging; AMH - Anti-Müllerian hormone; PEB - Cisplatin, Etoposide and Bleomycin; PEI - Cisplatin, Etoposide and Ifosfamide.

**Figure 4:**
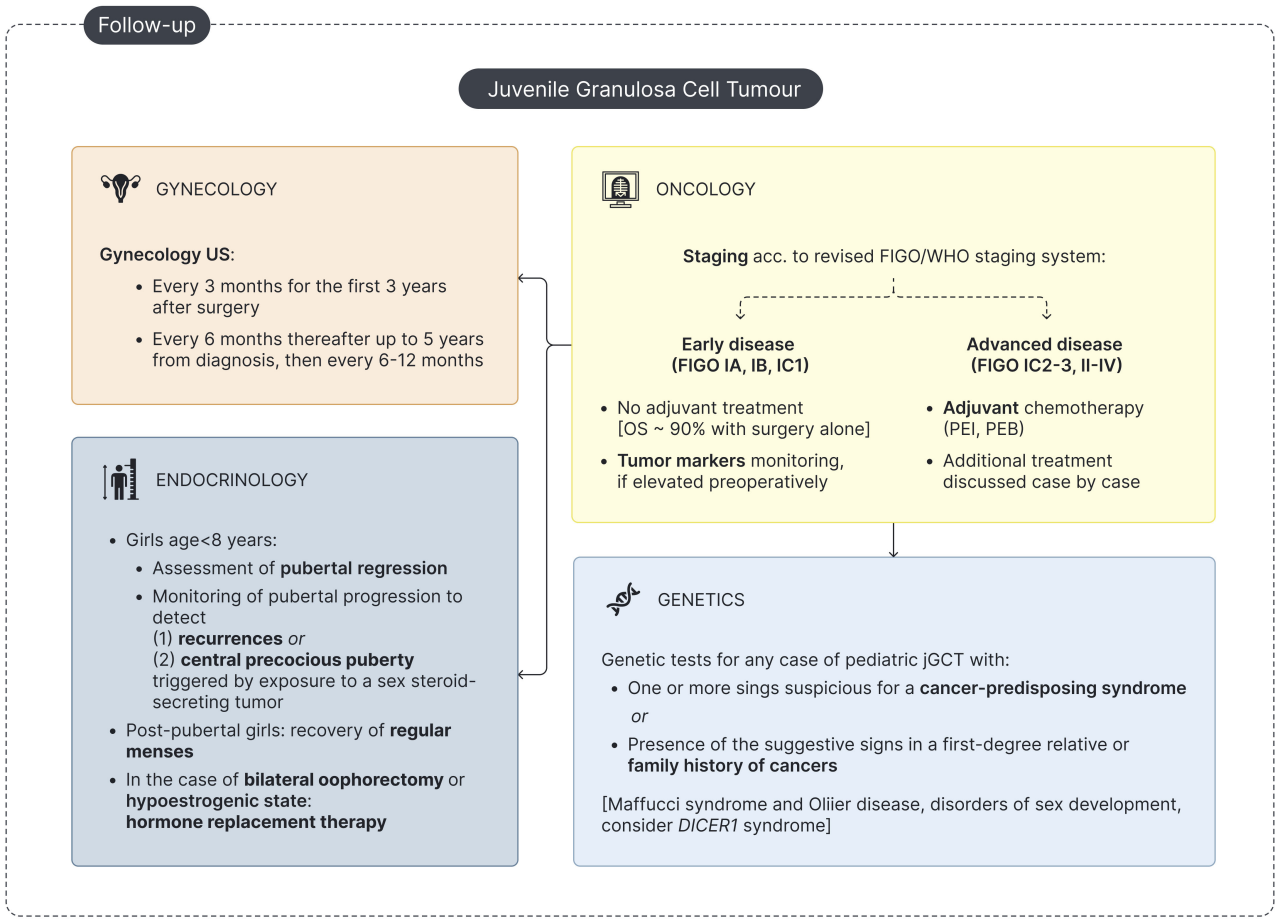
Flowchart for pediatric jGCT management at follow-up. 'US - Ultrasound; OS - overall survival; PEB - Cisplatin, Etoposide and Bleomycin; PEI - Cisplatin, Etoposide and Ifosfamide.

A comprehensive endocrine assessment is essential for the management of jGCT patients and should focus on (a) establishing an accurate differential diagnosis at onset (**Figure 3**) and (b) monitoring for the potential recurrence of signs of precocious puberty through clinical and biochemical follow-up (**Figure 4**).

### The role of the endocrinologist in the differential diagnosis of precocious puberty

Precocious puberty in girls is defined as the premature development of secondary sexual features before the age of 8 years (53). Central precocious puberty (CPP) is idiopathic in over 95% of female patients and represents the most common epidemiological condition (54, 55). CPP is also defined as “GnRH-dependent”, as it is the result of the premature activation of the hypothalamic–pituitary–gonadal axis, leading to early gonadotropin secretions and, accordingly, secondary ovarian stimulation (56, 57). Conversely, peripheral precocious puberty (PPP) is a rarer “GnRH-independent” cause of sexual precocity (8). It is the result of the precocious secretion of sex hormones by a peripheral ectopic source, often indicating an underlying malignancy (8). Differentiating between PPP and CPP is crucial, as management strategies differ significantly. Clinically, CPP is always isosexual, whereas PPP may present with either isosexual or heterosexual pubertal signs.

In this setting, hormonal tests play a central diagnostic role. The co-occurrence of elevated preoperative estradiol levels and suppressed LH and FSH prompts the diagnosis of PPP (58–61). Moreover, in patients with clinically detectable signs of virilization, elevated testosterone levels are generally detected (19, 59, 62). When a dynamic assessment is performed, a flat response of LH and FSH following the administration of gonadotropin-releasing hormone (GnRH stimulation test) corroborates the clinical suspicion of PPP.

When an adnexal mass is identified, second-level tests should include anti-Müllerian hormone (AMH) and inhibin A/B dosage. When assessed before surgery, inhibin A/B and AMH were constantly elevated in the previously published cases of jGCT (58, 61–64).

In addition, an unusual case of jGCT presented with acanthosis nigricans and hyperinsulinism, which resulted from peripheral insulin resistance driven by tumor-induced hyperandrogenemia and mimicking HAIR-AN syndrome (hyperandrogenism, insulin resistance, and acanthosis nigricans syndrome). The laboratory tests and clinical signs normalized following unilateral salpingo-oophorectomy (62).

Complementing clinical and laboratory assessments, radiological imaging plays a pivotal role in the diagnostic workup of girls with precocious puberty. Abdominal US is crucial in the initial evaluation of any girl presenting with PPP to detect pelvic or abdominal masses. Notably, jGCT can also arise in extraovarian sites, as exemplified by a case of adrenal gland localization (65). However, in patients with PPP, hypothalamic exposure to sex steroids may trigger the premature activation of the GnRH–gonadotropin–gonadal axis, finally leading to CPP as a consequence of the precocious GnRH-independent secretion of sexual hormones (12, 13, 56). Therefore, in selected cases of CPP, especially those with rapid progression or occurring at a younger age, abdominal US may be warranted to rule out underlying ovarian or adrenal pathology. Pelvic US plays a crucial role as a supplementary examination in the diagnostic workup of precocious puberty in girls, enabling the evaluation of estrogenization of the internal genitalia (66–68). Finally, preoperative MRI and histopathological examination are necessary for definitive diagnosis and staging of jGCT (**Figure 3**).

### Endocrine follow-up for patients with jGCT

A dedicated endocrine follow-up after treatment for jGCT is highly recommended (**Table 3**, **Figure 4**). Firstly, endocrine follow-up is meant to confirm the clinical and biochemical regression of PPP in pre-pubertal girls and to monitor the recovery of regular menstrual activity in post-pubertal girls experiencing primary amenorrhea/oligomenorrhea upon diagnosis. Secondly, physical examination and hormonal assessment is essential to timely detect signs consistent with disease recurrence or with the onset of CPP secondary to exposure to a sex-steroid-secreting tumor. Finally, hormone replacement therapy (HRT) may be required in selected cases (**Figure 4**).

**Table 3:** Clinical follow-up data of jGCT in the reviewed cohort.

Follow-up	n° of patients (%)
Endocrinology	
*Patients aged ≤ 8 years*	*57*
Central precocious puberty	3 (5.3%)
• Hypothalamic hamartoma	1 (1.7%)
• Secondary to sex-steroids secreting jGCT	2 (3.6%)
Transient isolated thelarche	1 (1.7%)
HRT for pubertal progression	1 (1.7%)
Genetics	
*All patients*	*94*
Any diagnosis of genetic or systemic condition	21 (22.3%)
• Ollier Disease	14 (14.9%)
• Maffucci syndrome	1
• Ovotesticular Disorder of Sex Development	1
• *DICER1* syndrome	1
• Beckwith-Wiedemann syndrome	1
• Li-Fraumeni and *PTEN* hamartoma tumor syndrome	1
• Neurofibromatosis type 1	1
• Tuberous sclerosis	1
Oncology	
*All patients*	*94*
Secondary malignancy	6 (6.4%)
• Contralateral metachronous jGCT	2 (2.1%)
• Low-grade chondrosarcoma	1
• Liposarcoma	1
• Ovarian mucinous cystadenoma	1
• Ovarian serous cystadenoma	1

Tumor excision with unilateral salpingo-oophorectomy in localized disease leads to progressive regression of PPP signs in prepubertal patients. Telarche, signs of estrogenization and, if present, virilization often require several months to achieve complete remission. Consistently, growth velocity shows a progressive decrease over time upon auxological monitoring.

The detection of precocious or rapidly progressing pubertal development in a previously treated prepubertal girl should raise suspicion for recurrence of jGCT or the onset of CPP secondary to sex steroid exposure. Accordingly, periodic hormonal tests during follow-up are recommended. Estradiol and testosterone may serve as tumor markers in jGCT when elevated preoperatively. However, a potential complication for prepubertal patients with jGCT is the development of CPP secondary to exposure to sex-steroid-secreting tumors (12, 13, 56). Priming of the hypothalamic–pituitary–gonadal axis by sex-steroid-secreting tumors has been described in two cases of jGCT and previously reported in several cases of congenital adrenal hyperplasia, adrenal tumors, germ cell tumors, McCune–Albright syndrome, and exposure to endocrine disruptors (12, 13, 54, 69–71) (**Table 3**). When clinical and biochemical signs consistent with pubertal re-flare are detected following jGCT treatment, a complete hormonal assessment is warranted, including baseline endocrine tests (FSH, LH, estradiol, and testosterone), GnRH stimulation test (if required), and abdominal US, which is mandatory to rule out jGCT recurrence. In addition, for patients with a biochemical profile suggestive of CPP, cranial MRI is recommended to exclude coexisting central neoplasms (56, 72).

Finally, clinical and biochemical follow-up by endocrinologists is essential to treat patients requiring HRT, either to induce pubertal progression in prepubertal girls or to manage the resulting hypoestrogenic state in post-pubertal girls. Ovarian jGCT may be hormone-dependent, and sex steroids serve as tumor markers postoperatively (73) so that HRT should be avoided immediately after surgery (74). In post-pubertal cases, the risk of cancer recurrence must be carefully balanced against the adverse effects of prolonged hypoestrogenism (74). Fertility-sparing surgery is mandatory in the management of jGCT in childhood even in cases of bilateral ovarian involvement; however, contralateral oophorectomy is indicated for patients with contralateral ovarian relapse or metachronous contralateral jGCT (5, 75). In these selected cases, long-term endocrinological follow-up should be planned for HRT. Some patients may require HRT after unilateral salpingo-oophorectomy if further treatments, particularly radiotherapy, were needed (76) (**Table 3**). Pediatric patients undergoing pubertal induction typically receive progressively increasing doses of estrogens over the first 2 to 3 years. Progestin is added in non-hysterectomized patients only once adequate uterine estrogenization and development have been achieved in order to mimic physiological pubertal development and induce menstruation (77, 78). The timing and regimen of HRT should be individualized, considering the patient’s age, clinical needs, any associated comorbidities, and the patient’s request for contraception (77, 78).

### The point of view of the gynecologist and the surgeon

Gynecologists or pediatric surgeons may be involved in the management of jGCT depending on the patient’s age and center-specific expertise. They play a crucial role in assessment at presentation, in follow-up, and in surgical procedure, which is essential for definitive diagnosis and pathological staging.

### Gynecological evaluation in pediatric jGCT

Gynecologists or pediatric surgeons are often the first specialists consulted in pediatric jGCT, either in elective or urgent/emergency settings (**Figure 3**). In addition, gynecologic US represents the first-line imaging modality for both diagnosis and follow-up, given its feasibility, non-invasiveness, and cost-effectiveness.

### Elective evaluation

In our review, some of the most reported symptoms at presentation were associated with mass effect in the pelvis or abdomen. Among the 94 patients included, 18.1% reported abdominal pain or discomfort at diagnosis, while 15.9% presented abdominal distension and 8.5% with constipation, anorexia, or sub-occlusive symptoms. Notably, the prevalence of mass-effect-related symptoms was higher among post-pubertal patients, with 66.7% experiencing at least one abdominal symptom at diagnosis. Additionally, Meigs syndrome was described in 2.1% presenting with increasing abdominal girth at onset, while 8.5% showed ascites secondary to tumor capsule rupture or malignant peritoneal involvement (**Table 2**).

### Urgent/emergency evaluation

In our review, 5.3% of patients with jGCT needed urgent surgery for acute abdomen at presentation to tumor rupture (2, 79) (**Table 2**).

### Diagnostic workup and disease monitoring

In the diagnostic workup of pediatric jGCT, gynecologists are involved to (a) evaluate internal genitalia estrogenization in prepubertal patients and (b) characterize the adnexal mass. Gynecologic US includes measuring the ovarian size, uterine size, uterine body-to-cervix ratio, and endometrial thickness (**Figure 3**) (66–68). Two pattern recognitions of GCT have been identified on gynecologic US: “pattern 1”, characterized by a solid mass with a heterogeneous echogenicity, and “pattern 2”, defined as a multilocular solid lesion with numerous small locules and a cystic component of mixed or low-level echogenicity, giving the mass a typical “Swiss cheese” appearance (80). Postoperative monitoring relies on pelvic/abdominal US to detect recurrences and assess pubertal regression by evaluating internal genitalia parameters in prepubertal patients (**Figure 4**) (66). On US, recurrent GCTs may exhibit two typical patterns: a small solid tumor and a tumor with vascularized echogenic ground-glass-like content (81). Finally, two girls were diagnosed prenatally in the setting of obstetric monitoring and presented at birth with virilization and ambiguous genitalia secondary to prenatal exposure to testosterone (19, 20).

### Surgical management of pediatric jGCT

The surgical management of jGCTs depends on the extent of the disease and the clinical context at the time of surgery—whether elective or emergency. The decision-making process should be individualized, considering the burden of disease and fertility preservation goals. Since jGCTs primarily affect children, adolescents, and young adults, fertility-sparing surgery is generally preferred, aiming to balance oncologic radicality with the preservation of ovarian function (10).

The treatment of choice for early-stage jGCT is unilateral salpingo-oophorectomy, but oophorectomy can be considered in tumors confined to the ovary, as recommended by the EXPeRT/PARTNER groups and FIGO Cancer Report 2021 (7, 10, 11, 82). The surgical procedure must be completed by staging, which includes inspection of the contralateral ovary, the peritoneal cavity, and the lymph nodes, with biopsy of any suspicious lesions or lymph nodes (5). Biopsies of the contralateral ovary are not routinely recommended, and lymph node biopsies can be omitted in FIGO I disease (7, 11, 82). This approach is supported by retrospective studies on patients with jGCT, which have demonstrated its safety in early-stage disease. In fact, jGCT confined to the ovary is associated with an excellent prognosis, with survival rates exceeding 90% following surgery alone (1, 10, 82). In contrast, tumorectomy is not generally recommended since ovarian-sparing mass excision has been associated with a higher risk of recurrence due to potential microscopic residual disease (7, 10, 11, 64, 82, 83). In some cases, jGCT with a cystic macroscopic appearance may initially be managed with elective ovarian cystectomy. However, the surgical plan for these patients can be completed after the anatomopathological diagnosis of jGCT (11, 61, 64, 84, 85). Additionally, laparoscopic restaging surgery is a feasible option for patients with incomplete staging at the initial surgery (86).

Recurrence after non-radical surgery and relapsed disease remain significant concerns (82). Salvage surgery may be indicated in cases of localized relapse when complete resection is feasible (2, 83, 86–88). However, in advanced-stage disease, surgical management aims for maximal cytoreduction when possible, but systemic chemotherapy is necessary (84). For locally advanced and disseminated disease, debulking surgery is not indicated, except in the context of palliative care (5). However, surgical specimens are essential for anatomopathological examination and are critical to guide chemotherapy treatment decisions (5). Moreover, obtaining anatomopathological specimens through surgery may also be crucial in cases of relapsed disease. Especially in syndromic patients, obtaining surgical specimens is essential to distinguish between tumor relapse and a second malignancy, which is crucial to define the appropriate treatment plan (14, 85, 89).

Remarkably, up to 10% of patients with jGCT may develop a contralateral metachronous ovarian tumor (84). In our review, 4.3% of patients with jGCT developed a contralateral ovarian tumor at a median follow-up of 2.3 years (range: 0.12–7 years) (19, 90–92). In cases of contralateral metachronous tumors with different histology, ovarian-sparing surgery is the preferred approach, when feasible. Accordingly, it was performed in two patients who developed a contralateral mucinous and serous cystadenoma (91, 92). However, in cases of contralateral metachronous jGCT, a second oophorectomy becomes a necessary therapeutic option (19, 90) (**Table 3**).

The possibility of contralateral ovarian secondary tumors requiring contralateral oophorectomy underscores the importance of addressing fertility preservation in jGCT patients. Although several fertility preservation techniques are available for cancer patients, in the context of ovarian cancer, oocyte and embryo cryopreservation appear to be the only viable options and can be proposed only for post-pubertal patients (93). In fact, while ovarian tissue cryopreservation has been extensively studied, the risk of reintroducing malignant cells upon reimplantation remains an unresolved concern in ovarian cancer patients, and no data are currently available on its safety in this population (93–95).

### The point of view of the oncologist

The oncologist plays a crucial role in disease staging, defining the treatment plan—particularly for advanced-stage and relapsed disease—and ensuring long-term follow-up for patients with jGCT.

### Staging and frontline treatment for jGCT in childhood

As previously described, complete pathological staging is essential for prognosis assessment and treatment planning (5, 82, 96). Most jGCTs, especially sex-steroid-secreting tumors, are diagnosed at early stages. In our review, 71.2% of the 66 patients with jGCT—for whom disease stage was specified—presented with FIGO/WHO I disease (**Supplementary Table S3**). Localized disease can be treated with surgery alone, with excellent survival rates exceeding 90% when complete resection is achieved (1, 10, 82). Coherently, 95.7% of 47 FIGO/WHO I patients in our review were reported alive at the last follow-up (**Supplementary Figure S1**). Disease stage at diagnosis and the absence of residual disease after surgery represent two of the most relevant prognostic factors for jGCT (82).

In 2014, the FIGO Oncology Committee revised the FIGO/WHO staging system for ovarian cancers. A key update in this classification was the subdivision of stage IC, distinguishing the timing and cause of capsule rupture as intraoperative (IC1), preoperative (IC2), or associated with positive cytology in ascitic or peritoneal washing samples (IC3) (97). While avoiding intraoperative capsule rupture (IC1) is desirable, its independent impact on prognosis remains uncertain, making the need for adjuvant chemotherapy in these cases questionable (5). Conversely, the finding of malignant cells in ascitic or peritoneal cytology (FIGO/WHO IC3) prompts the use of adjuvant chemotherapy (5). For spontaneous tumor rupture (FIGO/WHO IC2), the EXPeRT/PARTNER groups recommend adjuvant chemotherapy as well (5). However, in our retrospective review, three out of five FIGO/WHO IC2 patients did not receive adjuvant chemotherapy and were alive at follow-up with a range of 3–8 years, making indication for chemotherapy in this stage a questionable option (2, 71, 96) (**Supplementary Table S3**).

In cases of advanced-stage disease (FIGO/WHO stages II–IV), surgery alone is unlikely to achieve complete resection, and chemotherapy is necessary. Thus, 78.9% of 19 patients with FIGO/WHO stage II–IV disease received adjuvant chemotherapy (**Supplementary Table S3**). In selected cases with a biopsy-confirmed diagnosis, neoadjuvant chemotherapy may be considered prior to surgery (5).

Regarding overall survival, 92.7% of 69 patients with available outcome and follow-up data were reported to be alive, with a mean follow-up time of 4.8 years (range: 0.2–18 years). When focusing exclusively on patients with advanced-stage disease, 81.2% of 16 patients with complete follow-up data were alive at a mean follow-up of 4.4 years (range: 0.6–14 years) (**Supplementary Figure S1**). The mortality rate observed in advanced-stage patients in this review appears lower than previously reported based on recently published data from patients enrolled in the International Ovarian and Testicular Stromal Tumor and/or International Pleuropulmonary Blastoma/*DICER1* Registries (98). However, it is noteworthy that four patients (25.0%) with FIGO/WHO stage II–IV disease had a follow-up duration of less than 1.5 years. Given that the mean time to relapse in our review was 1.4 years (range: 0.12–8 years), this indicates that 25.0% of advanced-stage patients remained within the high-risk window for relapse at their last follow-up. Furthermore, among all patients with reported follow-up durations, 24.6% of 69 patients had a follow-up time of less than 1.5 years, representing a substantial proportion of patients whose short follow-up may have limited the possibility for recurrences to be observed.

Due to the rarity of jGCT, no prospectively generated treatment guidelines exist for advanced-stage disease. To standardize treatment regimens, the EXPeRT/PARTNER groups issued consensus recommendations in 2021 for the diagnosis and therapy of OSCSTs in children and adolescents, recommending cisplatin-based chemotherapy regimens, either PEB or PEI (7). Data on adults have demonstrated no superiority of either of these two regimens (75). Thus, the toxicity profile should be taken into consideration when defining treatment planning. PEB is less myelotoxic but carries a risk of pulmonary toxicity due to bleomycin, while PEI, in addition to being more myelotoxic, is associated with gonadotoxicity (75, 99). The recommendation is to administer four cycles of chemotherapy, with escalation to six cycles in cases of metastatic disease (5).

### Relapsed disease and survival

Relapse in jGCT remains a major concern for pediatric oncologists due to poor survival outcomes. In our review, 17.0% of the 94 patients included experienced one or more disease relapses, and 43.7% of these later ultimately died of disease. Relapse sites included the ipsilateral ovarian bed or abdomen/pelvis (*n* = 8), lymph nodes (*n* = 4), bone and bone marrow (*n* = 2), liver (*n* = 2), lungs (*n* = 1), omentum (*n* = 1), and peri-splenic region (*n* = 1).

Treatment for relapse involves a combination of chemotherapy and cytoreductive surgery (84). Several chemotherapy regimens, including PEB/PEI and VAC/VI (vincristine, dactinomycin, and cyclophosphamide alternating with vincristine and irinotecan), have been used at relapse, while paclitaxel, bevacizumab, gemcitabine, and thalidomide are typically considered as salvage therapy (84). For progressive disease, where prognosis remains extremely poor, additional treatment options have been explored, including radiotherapy (100), hyperthermic intraperitoneal chemotherapy with cisplatin in combination with cytoreductive surgery (85), and high-dose chemotherapy followed by autologous bone marrow transplantation (5, 76, 100). In addition, given the functional hormonal nature of granulosa cell tumors and their expression of sex steroid receptors, there is a rationale for a hormone-based approach. Limited data on adult patients support the use of hormone therapy with aromatase inhibitors as a viable treatment option for advanced-stage or recurrent GCT (75, 101).

### Oncologic long-term follow-up for patients with jGCT

jGCTs are characterized by slow growth and a tendency for late recurrence. In our review, 16 patients experienced disease relapse with a mean time to recurrence of 1.4 years (range: 0.12–8 years). The recommended surveillance for disease recurrence includes physical examination with endocrine assessment, monitoring of tumor markers if elevated preoperatively (AMH, inhibin A/B, estradiol, and/or testosterone), and pelvic/abdominal US. Notably, up to 10% of patients diagnosed with jGCT may develop a metachronous contralateral ovarian tumor over a wide follow-up period, ranging from weeks to 7 years, underlining the need for long-term follow-up for these patients (5, 19, 90–92). Follow-up evaluations should be conducted every 3 months during the first 3 years after diagnosis and may be spaced out thereafter (**Figure 4**) (5).

### The point of view of the geneticist

Previous molecular studies on large cohorts of pediatric cancers suggested that 10% of children diagnosed with cancer carry a predisposing germline variant (102–104). In addition, patients with cancer-predisposing syndromes (CPSs) present an increased lifetime risk to develop cancers and are known to be prone to treatment-induced second malignancies (105, 106). In this perspective, increasing awareness of CPSs is of utmost importance to refer families for genetic counseling (**Figure 4**) and to establish a surveillance plan for early tumor detection in affected patients when indicated (**Figure 3**).

#### Genetic counseling

The development of jGCT was previously described in (a) Ollier disease and Maffucci syndrome, (b) disorders of sex development, (c) *DICER1* syndrome, (d) *PTEN* hamartoma tumor syndrome and Li-Fraumeni syndrome, and (e) other genetic conditions (**Table 3**).

The association between jGCTs and Ollier disease (OD) (OMIM #166000) or Maffucci syndrome (MS) (OMIM #614569) has been recognized for decades. In our review, 15 patients with jGCT had a concurrent diagnosis of either Maffucci syndrome (*n* = 1) or Ollier disease (*n* = 14). In 10 (66.7%) of these cases, the skeletal disorder was diagnosed before the oncologic diagnosis, whereas in 5 patients (33.3%), radiologic investigations performed for jGCT incidentally revealed multiple enchondromas, raising suspicion for Ollier disease or Maffucci syndrome. When bone lesions are detected incidentally during radiologic staging for ovarian jGCT, histopathological examination may be required to distinguish enchondromas from bone metastases (2). These conditions are typically diagnosed within the first months of life due to bone deformities and pain, which are later followed by the identification of multiple enchondromas, either with (Maffucci syndrome) or without (Ollier disease) hemangiomas. The absence of a familial recurrence pattern is a key criterion for clinical diagnosis (107). Although these syndromes are not associated with a germline monogenic transmission, molecular analyses of jGCT and enchondroma tissues have identified somatic variants in *IDH1*, *IDH2*, and *PTH1R* genes, supporting the hypothesis of a diffuse mesenchymal dysfunction (108–110). In addition to their increased risk of developing jGCT compared to the general population, patients with Ollier disease and Maffucci syndrome are also predisposed to chondrosarcomas (either *de novo* or arising from enchondroma transformation) and gliomas. Furthermore, other malignancies have been reported in both disorders, including acute myeloid leukemia in Ollier disease and pancreatic or hepatic adenocarcinoma, astrocytoma, and various types of sarcomas in Maffucci syndrome (107).

Additionally, jGCTs have also been reported in patients with disorders of sex development (DSDs) (111). DSDs represent a group of rare conditions characterized by abnormalities in the development of internal and external genitalia. The risk of malignant transformation in dysgenetic gonads has been correlated with the presence of Y chromosome material, which predisposes individuals to gonadoblastoma and/or dysgerminoma (112). Therefore, the decision to proceed with gonadectomy should be individualized, and molecular characterization is essential to guide management (111).

In our review, we identified one case of mixed Sertoli–Leydig and jGCT in a *DICER1* patient (85). *DICER1* syndrome (OMIM #601200) is a dominantly inherited CPS caused by pathogenic variants in *DICER1* gene, which encodes a RNase III endonuclease involved in microRNA maturation. While this condition is transmitted through heterozygous germline variants, tumor development is typically associated with a second somatic mutation (second-hit) as observed in tumor tissues (14, 113), supporting *DICER*’s role as a tumor suppressor gene. Patients with *DICER1* syndrome are predisposed to various benign conditions, including lung cysts, cystic nephroma, multinodular goiter, juvenile hamartomatous intestinal polyps, and nasal chondromesenchymal hamartoma. Additionally, the affected individuals have an increased risk of developing multiple malignancies, such as pleuropulmonary blastoma, renal sarcoma, Wilms tumor, ciliary body medulloepithelioma, genitourinary embryonal rhabdomyosarcoma, pineoblastoma, and pituitary blastoma. The most frequently diagnosed malignancy in *DICER1* syndrome is Sertoli–Leydig cell tumor (SLCT); however, cases of gynandroblastomas with concurrent Sertoli and granulosa cell differentiation have also been reported (99, 114). In addition, SLCT in patients with *DICER1* syndrome can be misclassified as jGCTs (115). While the European Society of Gynecological Oncology (ESGO)/International Society of Pediatric Oncology Europe (SIOPE) guidelines state that current evidence is insufficient to recommend genetic testing for *DICER1* syndrome in OSCSTs of the jGCT subtype, anatomopathological studies have documented *DICER1* pathogenic variants in cases of jGCT (85, 98, 99, 115). The correlation of clinical and anatomopathological data suggests that the presence of *DICER1* variants in OSCSTs may have prognostic significance rather than serving to differentiate histopathological subtypes (85, 99, 115). Finally, a significant proportion of patients with *DICER1* syndrome present with overgrowth syndrome and/or macrocephaly (14, 15), which was the clinical feature that prompted genetic testing in our patient, though mutations involving this gene are rarely associated with jGCT.

One patient with jGCT was diagnosed with Li–Fraumeni syndrome (LFS) (OMIM #609265) and *PTEN* hamartoma tumor syndrome (PHTS) (OMIM #158350), both of which are dominantly inherited CPSs (88). PHTS encompasses multiple syndromic conditions characterized by a broad spectrum of clinical features. The affected individuals have a significantly increased lifetime risk of breast, endometrial, and colorectal cancer as well as renal cell carcinoma and melanoma; however, thyroid cancer represents the most common malignancy risk during childhood (15). Clinical manifestations such as overgrowth syndrome and/or macrocephaly may further prompt genetic testing for these conditions.

Finally, we highlight three patients with jGCT who were diagnosed with Beckwith–Wiedemann syndrome (OMIM #130650) (116), tuberous sclerosis complex (OMIM #191100) (117), and neurofibromatosis type 1 (OMIM #162200) (118). These genetic disorders are already known to predispose individuals to specific malignant and benign tumors. However, to date, there is no evidence suggesting an increased risk of jGCT in these conditions.

### Surveillance plan for early tumor detection in CPSs

Notably, both the *DICER1* patient (85) and the LFS/PHTS patient (88) included in our review faced a tumor-related death. Prospective observational studies on cancer surveillance in LFS have demonstrated that a combination of clinical, biochemical, and radiologic follow-up can improve long-term survival. These findings highlight the importance of raising awareness of CPSs and prospectively evaluating surveillance protocols for affected patients (119, 120). Additionally, as previously described, early-stage jGCT is associated with better prognosis and overall survival (1, 10). These findings support the rationale to establish a structured cancer surveillance plan for patients diagnosed with other CPSs. In this context, the International *DICER1* Symposium has established consensus-based surveillance recommendations for *DICER1*-associated tumors (14, 15), followed by the more recent recommendations provided by the Host Genome Working Group (HGWG) of the SIOPE and the Clinical Guideline Working Group of the CanGene (114). Similarly, the National Comprehensive Cancer Network (NCCN) has issued guidelines for thyroid cancer monitoring in children and cancer risk surveillance in adults with PHTS, while the PHTS Guideline Development Group and the European Reference Network GENTURIS introduced updated surveillance recommendations in 2020 (121, 122). However, prospective studies evaluating the efficacy of surveillance plans in improving overall survival for patients with *DICER1* syndrome and PHTS are still lacking and would be essential to develop evidence-based protocols (114). Similarly, while there is clear evidence of malignant transformation of enchondromas in OD and MS, as well as a known predisposition to glioma development, there are no evidences supporting that a cancer surveillance plan could improve the survival rates for these patients, especially considering that the tissue distribution and load of the *IDH* variant may vary significantly among affected individuals, which is likely to influence the risk of malignancy (123, 124).

While early tumor detection may be proven beneficial, a major challenge in improving survival outcomes for patients with CPSs is their high risk of developing treatment-induced second malignancies (105, 106). Therefore, identifying targeted therapies tailored to specific genetic mutations would be crucial in reducing the risk of secondary cancers in these patients. In this perspective, precision medicine treatment may ultimately prove meaningful in improving long-term survival rates for individuals affected with CPSs.

## Summary

Like other rare conditions, ovarian jGCTs represent a significant challenge for clinicians. Through a clinical case and a comprehensive review of the literature, we have highlighted the key role of a multidisciplinary approach to this rare pediatric cancer. We proposed a flowchart for the multidisciplinary management of jGCT at diagnosis and during follow-up, emphasizing the need for patient-centered care that integrates the work of oncologists, endocrinologists, surgeons, gynecologists, and geneticists while advocating for long-term survivorship care. Our contribution aims to provide a clinically applicable outline for the management of jGCT in childhood.
