# Supplementary material for: Management of ovarian granulosa cell tumor in childhood: a case report and recommendations for a multidisciplinary approach
Source: Front Oncol. 2025 Nov 24;15:1634166. doi: 10.3389/fonc.2025.1634166 (PMC12682638; doi:10.3389/fonc.2025.1634166)
Supplement: Supplementary file 1 [file DataSheet1.docx]

**Supplementary**

**Clinical case**

**Supplementary Data**

The NGS panel for overgrowth syndromes performed for the patient described in the clinical case included the following genes: *AKT1*, *AKT2*, *AKT3*, *APC2*, *BRWD3*, *CDKN1C*, *CHD8*, *DICER1*, *DIS3L2*, *DNMT3A*, *EED*, *EZH2*, *GPC3*, *GPC4*, *HIST1H1E*, *HRAS*, *MTOR*, *NFIX*, *NSD1*, *OFD1*, *PDGFRB*, *PIK3CA*, *PPP2R5D*, *PTEN*, *RNF125*, *SETD2*, *TCF20*, *WT1* and *KDM6B*.

**
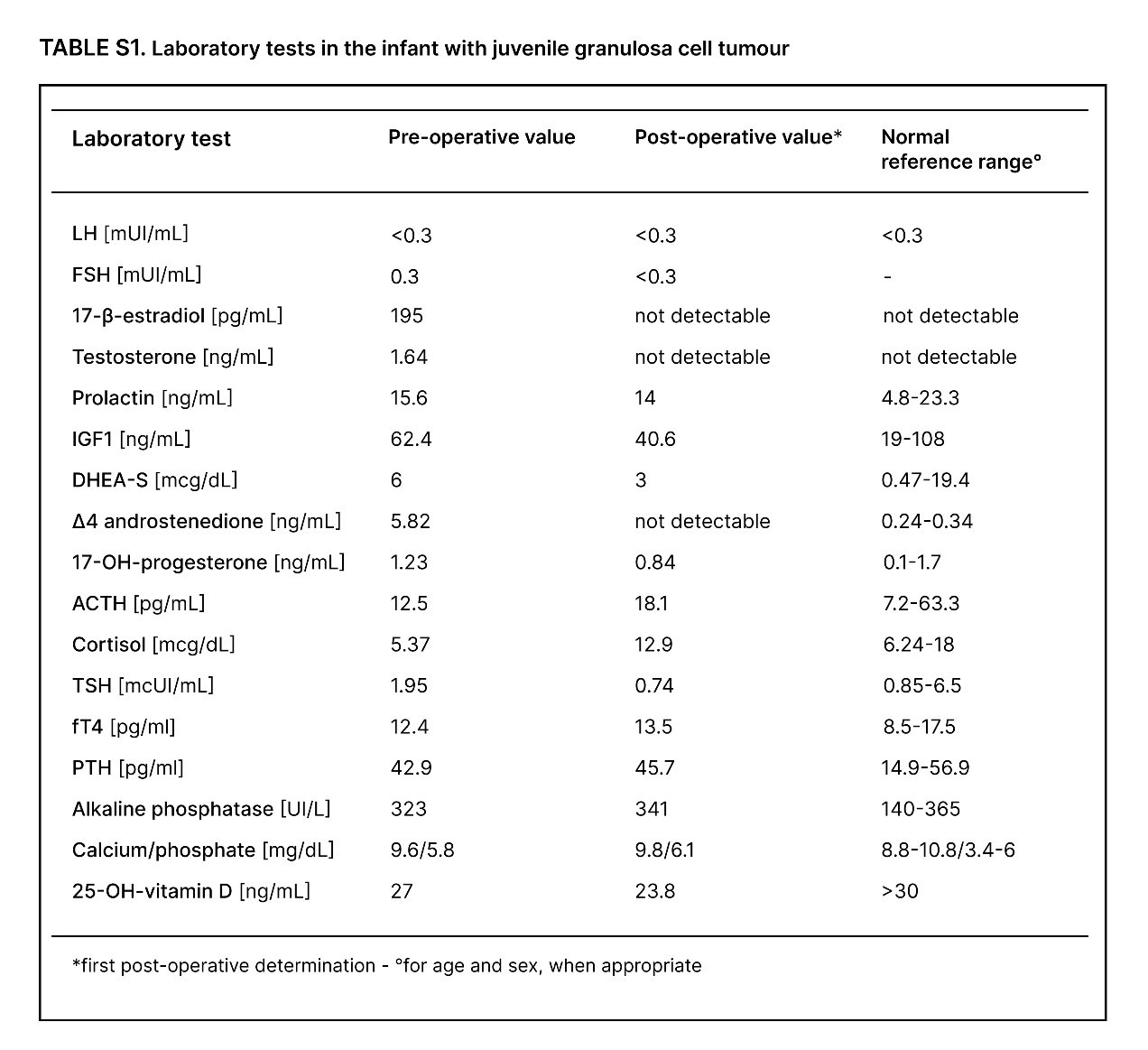
**

**Table S1. Laboratory tests in the infant with jGCT.**

**
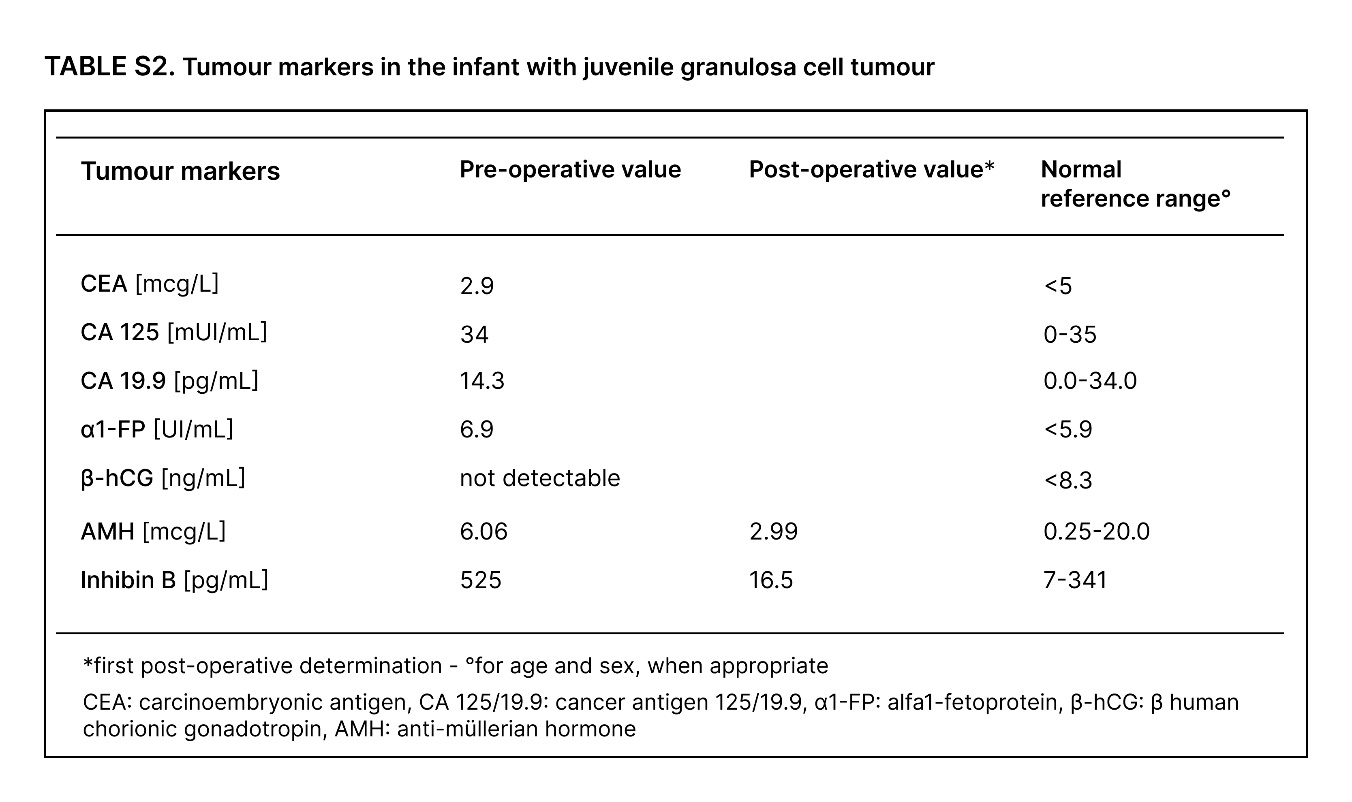
Table *S*2. Tumor markers in the infant with jGCT.**

**Review**

**
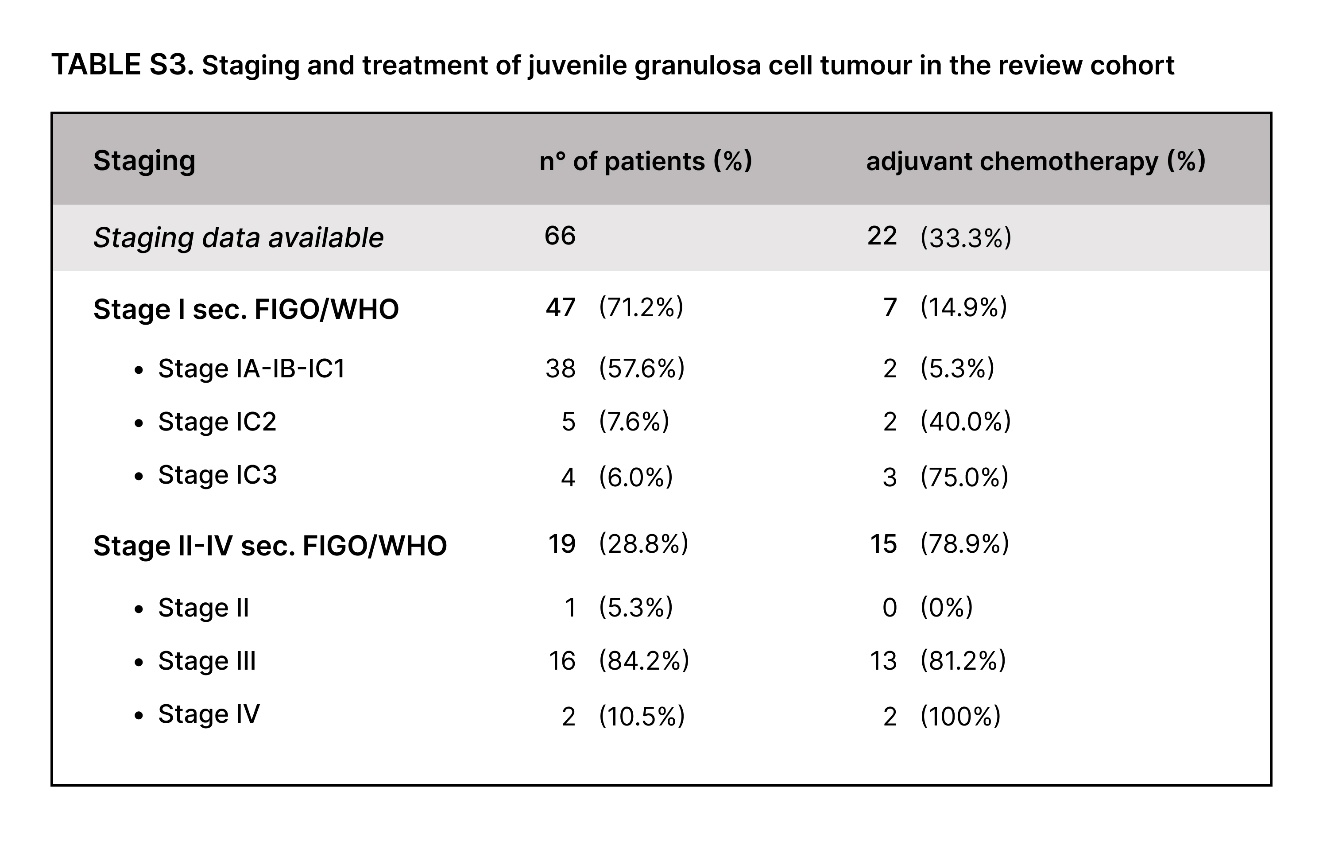
**

**Table S3. Table summarizing staging and chemotherapy treatment for the jGCT cases included in the retrospective review.**

Of the total 94 cases included, staging at diagnosis was available for 66. The percentage of patients who received adjuvant chemotherapy is reported relative to the total number of patients in each stage.

**
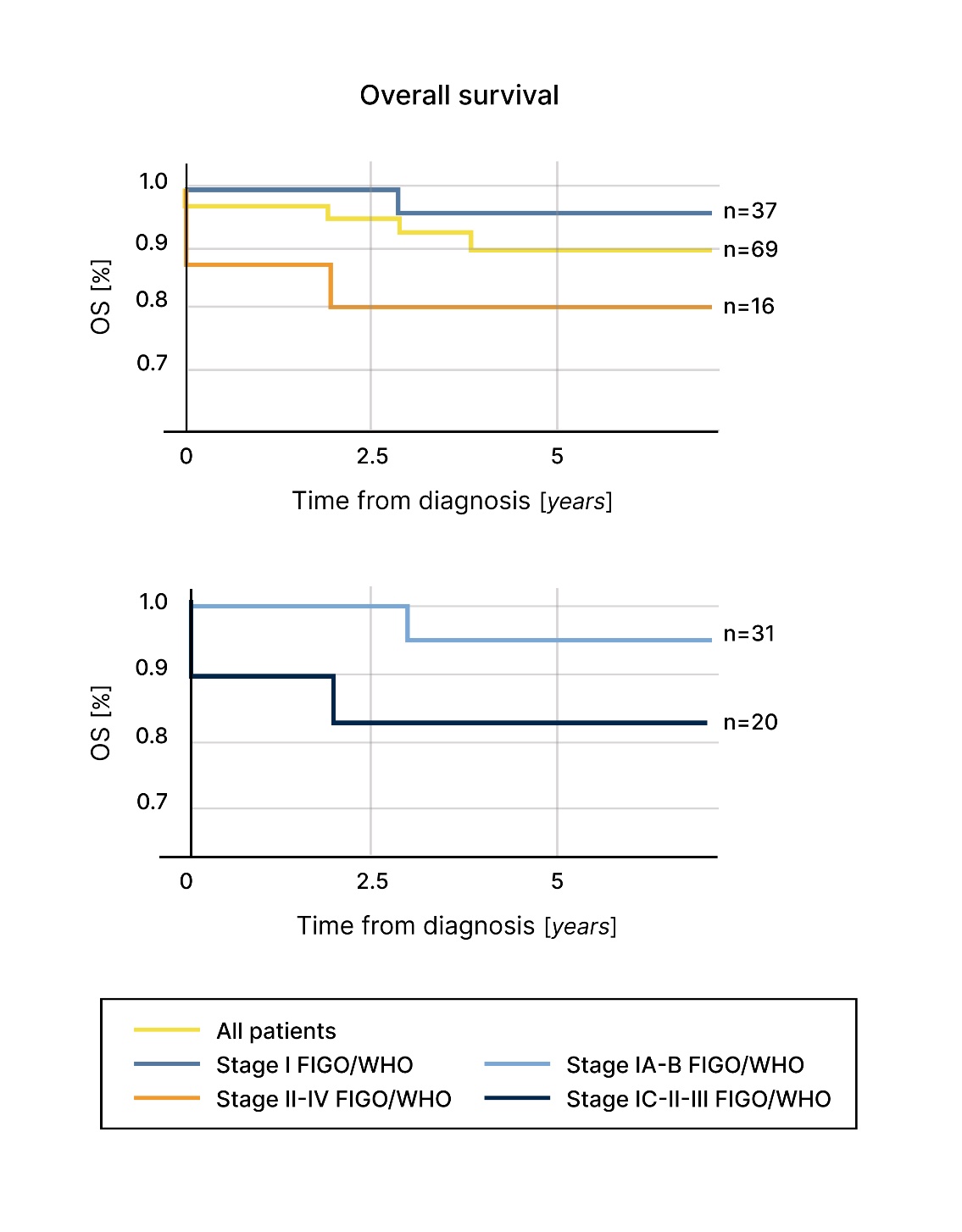
**

**Figure S1. Kaplan–Meier curves showing overall survival of the cohort of pediatric patients with a diagnosis of jGCT identified through literature review**.

*Graph above*. Overall survival - defined as the time from diagnosis to death from any cause or to censored data - is displayed. Among the 94 cases identified through the literature review, complete survival data and time to last follow-up or death from any cause were available for 69 patients (*yellow curve*). Of these, 53 patients had complete staging information: 37 were diagnosed with Stage I disease according to the FIGO/WHO classification (*blue curve)* and 16 with Stage II–IV disease (*orange curve*).

*Graph below*. Overall survival curves for patients with localized disease (Stage IA-B according to FIGO/WHO, *light blue curve*) and those with locally advanced disease (Stage IC-II-III according to FIGO/WHO, *dark blue curve*) are shown separately in the graph below. Both patients included in the review who had FIGO/WHO Stage IV disease at diagnosis deceased.
