# Supplementary material for: Management of ovarian granulosa cell tumor in childhood: a case report and recommendations for a multidisciplinary approach
Source: Front Oncol. 2025 Nov 24;15:1634166. doi: 10.3389/fonc.2025.1634166 (PMC12682638; doi:10.3389/fonc.2025.1634166)
Supplement: Supplementary file 2 [file Table1.docx]

**Table S3. Summary table of the clinical characteristics, presenting symptoms and frontline treatments of the pediatric patients with jGCT included in the review.**

| **Article identification** | | | | **Patients and diagnosis** | | | | | | **Genetics** | **Frontline treatment** | | |  |
| --- | --- | --- | --- | --- | --- | --- | --- | --- | --- | --- | --- | --- | --- | --- |
| **#** | **Articles** | **YOP** | **Pt n°** | | **N° cases** | **Age at diagnosis** [*years*] | **Endocrinologic disturbances at onset** | **Mass effect symptoms at onset** | **Stage at diagnosis** | **Diagnosis of genetic/other conditions** | **Surgery** | **Adjuvant chemo/other treatment** | **Type of adjuvant chemotherapy** | |
| **1** | Tamimi HK et al. | 1984 | **1** | | 1 | 15 | amenorrhea | increasing abdominal girth | NS | Ollier disease | unilateral salpingo-oophorectomy | adjuvant chemotherapy (refused) |  | |
| **2** | Pounder DJ et al. | 1985 | **2** | | 1 | 15 | irregular menses | palpable abdominal mass | NS | Ollier disease | unilateral oophorectomy | adjuvant chemotherapy | melphalan | |
| **3** | Colombo N et al. | 1986 | **3** | | 1 | 7 | NS |  | FIGO IV |  | unilateral salpingo-oophorectomy | adjuvant chemotherapy | 3 cycles of PVB | |
| **4** | Vaz RM et al. | 1986 | **4** | | 1 | 8,5 | PPP |  | NS | Ollier disease | unilateral salpingo-oophorectomy |  |  | |
| **5** | Schwartz HS et al. | 1987 | **5** | | 1 | 17 | none |  | NS | Ollier disease | NS |  |  | |
| **6** | Vassal G et al. | 1988 | **6** | | 15 | 1,8 | PPP |  | FIGO III |  | unilateral salpingo-oophorectomy | none |  | |
|  |  |  | **7** | |  | 2 | none |  | FIGO III |  | unilateral salpingo-oophorectomy | adjuvant chemotherapy and RT |  | |
|  |  |  | **8** | |  | 3,4 | PPP |  | FIGO III |  | bilateral salpingo-oophorectomy | none |  | |
|  |  |  | **9** | |  | 4 | PPP |  | FIGO I |  | unilateral salpingo-oophorectomy | none |  | |
|  |  |  | **10** | |  | 5,4 | PPP |  | FIGO I |  | unilateral salpingo-oophorectomy | none |  | |
|  |  |  | **11** | |  | 6 | PPP |  | FIGO I |  | unilateral salpingo-oophorectomy | none |  | |
|  |  |  | **12** | |  | 7,7 | PPP |  | FIGO II |  | bilateral salpingo-oophorectomy | none |  | |
|  |  |  | **13** | |  | 9,3 | none |  | NS |  | unilateral salpingo-oophorectomy | none |  | |
|  |  |  | **14** | |  | 10 | none |  | FIGO III |  | unilateral salpingo-oophorectomy | adjuvant chemotherapy |  | |
|  |  |  | **15** | |  | 10,8 | none |  | FIGO III |  | unilateral salpingo-oophorectomy | adjuvant chemotherapy |  | |
|  |  |  | **16** | |  | 11,5 | none |  | FIGO I |  | unilateral salpingo-oophorectomy | adjuvant chemotherapy |  | |
|  |  |  | **17** | |  | 12,3 | none |  | FIGO III |  | unilateral salpingo-oophorectomy | adjuvant chemotherapy |  | |
|  |  |  | **18** | |  | 13,9 | none |  | FIGO I |  | unilateral salpingo-oophorectomy | none |  | |
|  |  |  | **19** | |  | 15,4 | none |  | FIGO IV |  | bilateral salpingo-oophorectomy, hysterectomy | adjuvant chemotherapy | MAC, vincristine, bleomycin | |
|  |  |  | **20** | |  | 15,6 | virilization |  | FIGO I |  | unilateral salpingo-oophorectomy | none |  | |
| **7** | Velasco-Oses A et al. | 1988 | **21** | | 1 | 6 | PPP |  | FIGO IA | Ollier disease | unilateral salpingo-oophorectomy (urgency) |  |  | |
| **8** | Asirvatham R et al. | 1991 | **22** | | 1 | 4 | PPP |  | NS | Ollier disease | unilateral oophorectomy |  |  | |
| **9** | Le Gall C et al. | 1991 | **23** | | 1 | 12 | none | ascites | NS | Ollier disease | unilateral salpingo-oophorectomy |  |  | |
| **10** | Tanaka Y et al. | 1992 | **24** | | 1 | 15 | none | ascites | FIGO IC(3#) | Maffucci syndrome | unilateral salpingo-oophorectomy | adjuvant chemotherapy | cisplatin, doxorubicin | |
| **11** | Powell JL et al. | 1993 | **25** | | 1 | 13 | none (diagnosis during pregnancy) |  | FIGO IIIB |  | unilateral oophorectomy; post-partum surgery restaging with partial omentectomy | adjuvant chemotherapy | 3 cycles of methotrexate, actynomic D and chlorambucil | |
| **12** | Wessalowski R et al. | 1995 | **26** | | 1 | 3 | none | acute abdomen (rupture) | FIGO IC(2#) |  | unilateral oophorectomy | adjuvant chemotherapy | 5 cycles of ifosfamide, actinomycin D, vincristine, adriamycine | |
| **13** | Kukuvitis A et al. | 1995 | **27** | | 1 | 0,6 | PPP | ascites, anorexia and vomiting | FIGO IC(2#) |  | mass excision | none |  | |
| **14** | Silverman LA et al. | 1996 | **28** | | 2 | 2 | PPP and pubarche | palpable abdominal mass | NS |  | mass excision |  |  | |
|  |  |  | **29** | |  | 7,6 | PPP and pubarche | palpable abdominal mass | NS |  | mass excision |  |  | |
| **15** | Powell JL et al. | 1996 | **30** | | 2 | 13 | none | abdominal pain, Meigs syndrome | FIGO IIIC |  | unilateral oophorectomy | adjuvant chemotherapy | 6 cycles of carboplatin, VP16 | |
|  |  |  | **31** | |  | 17 | none | constipation, increasing abdominal girth | FIGO IIIC |  | unilateral salpingo-oophorectomy | adjuvant chemotherapy | 7 cycles of carboplatin, VP16 | |
| **16** | Bouffet E et al. | 1997 | **32** | | 3 | 1,3 | PPP, virilization, pubarche and axillarche | palpable abdominal mass | NS |  | unilateral salpingo-oophorectomy |  |  | |
|  |  |  | **33** | |  | 1,3 | PPP, virilization, pubarche | palpable abdominal mass | NS |  | unilateral salpingo-oophorectomy |  |  | |
|  |  |  | **34** | |  | 0,4 | none | palpable abdominal mass, ascites | FIGO III |  | unilateral oophorectomy | NS |  | |
| **17** | Feilberg Jørgensen N et al. | 1998 | **35** | | 1 | 1,5 | PPP |  | NS |  | unilateral oophorectomy |  |  | |
| **18** | Gell JS et al. | 1998 | **36** | | 1 | 13 | irregular menses | abdominal discomfort, increasing abdominal girth, palpable abdominal mass | NS | Ollier disease | unilateral salpingo-oophorectomy |  |  | |
| **19** | Daubenton JD et al. | 2000 | **37** | | 1 | 11 | none | abdominal pain, constipation, anorexia, palpable abdominal mass | FIGO III |  | NS | adjuvant chemotherapy | 6 courses of PEB | |
| **20** | Powell JL et al. | 2001 | **38** | | 1 | 17 | none | constipation, increasing abdominal girth, ascites | FIGO IIIC |  | unilateral salpingo-oophorectomy | adjuvant chemotherapy | 6 cycles of carboplatin, VP16 | |
| **21** | Erdreich-Epstein A et al. | 2002 | **39** | | 1 | 10,5 | none | abdominal pain | NS |  | unilateral oophorectomy |  |  | |
| **22** | Chan LF et al. | 2004 | **40** | | 1 | 3,9 | PPP, pubarche and axillarche | abdominal pain, palpable abdominal mass | NS |  | unilateral salpingo-oophorectomy |  |  | |
| **23** | Tenorio Romojaro V et al. | 2004 | **41** | | 1 | 1,3 | PPP |  | FIGO IA |  | unilateral salpingo-oophorectomy |  |  | |
| **24** | Koksal Y et al. | 2004 | **42** | | 1 | 11 | galactorrhea | palpable abdominal mass | FIGO IA |  | NS |  |  | |
| **25** | Kdous M et al. | 2004 | **43** | | 1 | 6 | PPP, virilization, pubarche and axillarche | palpable abdominal mass | FIGO I |  | unilateral salpingo-oophorectomy |  |  | |
| **26** | Till H et al. | 2005 | **44** | | 1 | 6 | PPP | palpable abdominal mass | FIGO IA |  | unilateral salpingo-oophorectomy |  |  | |
| **27** | Larizza D et al. | 2006 | **45** | | 1 | 16 | secondary amenorrhea |  | FIGO IA |  | unilateral oophorectomy |  |  | |
| **28** | Kdous M et al. | 2006 | **46** | | 1 | 6 | PPP | palpable abdominal mass | FIGO IA |  | unilateral oophorectomy |  |  | |
| **29** | Guo H et al. | 2006 | **47** | | 1 | 8 | PPP and pubarche | increasing abdominal girth | FIGO I | tuberous sclerosis | unilateral salpingo-oophorectomy | none |  | |
| **30** | Leyva-Carmona M et al. | 2009 | **48** | | 2 | 0,8 | PPP | increasing abdominal girth | FIGO IB |  | bilateral salpingo-oophorectomy |  |  | |
|  |  |  | **49** | |  | 0 | none | abdominal mass causing respiratory distress | FIGO IC(2#) | Ollier disease | urgent surgery for tumour rupture | none |  | |
| **31** | Pascual J et al. | 2009 | **50** | | 1 | 0,2 | ambiguous genitalia, virilization |  | NS | ovotesticular DSD | bilateral gonads excision |  |  | |
| **32** | Capito C et al. | 2009 | **51** | | 1 | 0 | ambiguous genitalia, virilization |  | NS |  | tumorectomy |  |  | |
| **33** | Hashemipour M et al. | 2010 | **52** | | 1 | 6 | PPP and pubarche | abdominal pain, nausea | NS |  | unilateral oophorectomy |  |  | |
| **34** | Wang Y et al. | 2011 | **53** | | 3 | 0,7 | none | palpable abdominal mass, abdominal distension | FIGO IA |  | NS |  |  | |
|  |  |  | **54** | |  | 3 | PPP and pubarche | palpable abdominal mass, ascites | FIGO IA |  | NS |  |  | |
|  |  |  | **55** | |  | 4 | none | abdominal pain | FIGO IC(2#) |  | unilateral salpingo-oophorectomy | none |  | |
| **35** | Paternoster M et al. | 2011 | **56** | | 1 | 0,8 | PPP | vomit, acute abdomen with hemoperitoneum | NS |  | post-mortem diagnosis |  |  | |
| **36** | Calcaterra V et al. | 2013 | **57** | | 1 | 8,4 | Vaginal bleeding in CPP in GnRH analogue | palpable abdominal mass | FIGO IA |  | NS |  |  | |
| **37** | Haroon NN et al. | 2013 | **58** | | 1 | 7 | PPP |  | NS |  | unilateral salpingo-oophorectomy (urgency, ovarian torsion) |  |  | |
| **38** | Bedir R et al. | 2014 | **59** | | 1 | 10 | none | abdominal distention, acute abdomen, ascites | FIGO IIIB |  | unilateral salpingo-oophorectomy | adjuvant chemotherapy | NS | |
| **39** | Dhivyalakshmi J et al. | 2014 | **60** | | 1 | 0,7 | pubarche |  | FIGO IA |  | unilateral salpingo-oophorectomy |  |  | |
| **40** | Benesch M et al. | 2015 | **61** | | 1 | 4,5 | PPP | abdominal pain | FIGO IA |  | mass excision | adjuvant chemotherapy | PEI | |
| **41** | Lamas-Pinheiro R et al. | 2016 | **62** | | 1 | 3 | PPP |  | FIGO IA | Beckwith-Wiedemann syndrome | unilateral salpingo-oophorectomy |  |  | |
| **42** | Sampagar AA et al. | 2016 | **63** | | 1 | 2,5 | PPP | palpable abdominal mass | FIGO IC3 | Ollier disease | unilateral salpingo-oophorectomy | adjuvant chemotherapy | NS | |
| **43** | Lacourt P et al. | 2017 | **64** | | 1 | 0,8 | PPP | palpable abdominal mass | NS |  | unilateral salpingo-oophorectomy |  |  | |
| **44** | Drucker NA et al. | 2017 | **65** | | 1 | 0 | ambiguous genitalia, virilization | palpable abdominal mass | FIGO IB |  | bilateral salpingo-oophorectomy |  |  | |
| **45** | Wu H et al. | 2017 | **66** | | 8 | 1,4 | PPP | abdominal distension | FIGO I | Li-Fraumeni and *PTEN* hamartoma tumor syndrome | unilateral salpingo-oophorectomy |  |  | |
|  |  |  | **67** | |  | 2,6 | PPP |  | FIGO I |  | unilateral salpingo-oophorectomy |  |  | |
|  |  |  | **68** | |  | 3,3 | PPP |  | FIGO I |  | unilateral salpingo-oophorectomy |  |  | |
|  |  |  | **69** | |  | 12,8 | none | abdominal distension | FIGO I |  | unilateral salpingo-oophorectomy |  |  | |
|  |  |  | **70** | |  | 0,9 | PPP |  | FIGO IA |  | unilateral salpingo-oophorectomy |  |  | |
|  |  |  | **71** | |  | 14,3 | none | abdominal distension | FIGO IIIB |  | unilateral salpingo-oophorectomy | adjuvant chemotherapy | 4 cycles of PEB; 5 cycles of paclitaxel/carboplatin; 3 cycles of Karenitecin; palliative chemotherapy with VP-16, ifosfamide and with paclitaxel and carboplatin | |
|  |  |  | **72** | |  | 0,7 | PPP | abdominal distension | FIGO III |  | unilateral salpingo-oophorectomy | adjuvant chemotherapy | BEP | |
|  |  |  | **73** | |  | 9,2 | none | palpable abdominal mass | FIGO III |  | unilateral salpingo-oophorectomy | adjuvant chemotherapy | vincristine, daptomycin, cyclophosphamide | |
| **46** | Mohapatra A et al. | 2019 | **74** | | 1 | 5 | PPP | increasing abdominal girth, uterine torsion | FIGO IA |  | unilateral salpingo-oophorectomy |  |  | |
| **47** | Andreetta M et al. | 2020 | **75** | | 1 | 5,8 | PPP |  | FIGO IA |  | unilateral oophorectomy |  |  | |
| **48** | Okawa ER et al. | 2020 | **76** | | 1 | 15,8 | amenorrhea/irregular menses | abdominal discomfort | NS |  | unilateral salpingo-oophorectomy |  |  | |
| **49** | Kwiatkowska A et al. | 2020 | **77** | | 1 | 17 | amenorrhea/irregular menses |  | FIGO IA |  | tumorectomy | adjuvant chemotherapy (refused) |  | |
| **50** | Jalaeefar A et al. | 2020 | **78** | | 1 | 17 | amenorrhea/irregular menses | abdominal distension, early satiety, anorexia, abdominal pain | FIGO IA | Ollier disease | unilateral oophorectomy |  |  | |
| **51** | Ben David Y et al. | 2021 | **79** | | 1 | 0 | ambiguous genitalia, virilization | Meigs syndrome | FIGO IA |  | urgent surgery for ovarian torsion |  |  | |
| **52** | Viet LA et al. | 2021 | **80** | | 1 | 4 | PPP | palpable abdominal mass | FIGO IC(2#) |  | NS | adjuvant chemotherapy | NS | |
| **53** | Barakizou H et al. | 2021 | **81** | | 1 | 2,2 | PPP |  | FIGO IA |  | urgent surgery for ovarian torsion |  |  | |
| **54** | De Sanctis V et al. | 2021 | **82** | | 1 | 0,8 | PPP |  | FIGO IA |  | unilateral salpingo-oophorectomy |  |  | |
| **55** | Hovsepyan S et al. | 2021 | **83** | | 2 | 16 | amenorrhea/irregular menses | palpable abdominal mass, ascites | FIGO IC(3#) |  | mass excision | adjuvant chemotherapy | 4 cycles of PEI | |
|  |  |  | **84** | |  | 15 | amenorrhea/irregular menses |  | FIGO IC(3#) |  | tumorectomy | none |  | |
| **56** | Gaikwad et al. | 2022 | **85** | | 1 | 5.4 | PPP |  | NS |  | mass excision | adjuvant chemotherapy | PEB | |
| **57** | Qutub LM et al. | 2022 | **86** | | 1 | 6 | PPP | abdominal pain, abdominal, palpable abdominal mass | NS | Neurofibromatosis type 1 | NS |  |  | |
| **58** | Kim RC et al. | 2022 | **87** | | 1 | 4 | virilization |  | FIGO IA |  | NS |  |  | |
| **59** | Khatun F et al. | 2022 | **88** | | 1 | 4 | PPP | palpable abdominal mass | NS |  | NS |  |  | |
| **60** | Littrell LA et al. | 2023 | **89** | | 1 | 15 | amenorrhea/irregular menses | abdominal pain, early satiety | FIGO IA | Ollier disease | NS |  |  | |
| **61** | Zhang J et al. | 2023 | **90** | | 1 | 4 | PPP |  | FIGO IA | Ollier disease | NS |  |  | |
| **62** | Park H et al. | 2023 | **91** | | 1 | 12 | amenorrhea/irregular menses | abdominal pain | FIGO IA |  | NS |  |  | |
| **63** | Devins KM et al. | 2024 | **92** | | 1 | 10 | none |  | NS | Ollier disease | NS |  |  | |
| **64** | Shero N et al. | 2024 | **93** | | 1 | 14 | none | abdominal pain, nausea and vomiting | NS | *DICER1* syndrome | ovarian cystectomy, revision with unilateral salpingo-oophorectomy | none |  | |
| **65** | Amirkashani D et al. | 2024 | **94** | | 1 | 6 | PPP | abdominal pain, abdominal distention, nausea | NS |  | unilateral salpingo-oophorectomy |  |  | |
|  |  |  |  | |  |  |  |  |  |  |  |  |  | |

[YOP – year of publication; NS – not specified; PPP – peripheral precocious puberty; RT – radiotherapy; PVB – cisplatin, vinblastine and bleomycin; MAC – mitomycin C, adriamycin and cyclophosphamide; PEB – cisplatin, etoposide and bleomycin; PEI – cisplatin, etoposide and ifosfamide; (-#) added by author]
