# Supplementary material for: Management of ovarian granulosa cell tumor in childhood: a case report and recommendations for a multidisciplinary approach
Source: Front Oncol. 2025 Nov 24;15:1634166. doi: 10.3389/fonc.2025.1634166 (PMC12682638; doi:10.3389/fonc.2025.1634166)
Supplement: Supplementary file 4 [file Table3.docx]

**Table S5. Summary table of follow-up and outcomes of the pediatric patients with jGCT included in the review.**

| **Article identification** | | | | **Patients** | | | **Follow-up** | | | | | | **Outcome** | |  |
| --- | --- | --- | --- | --- | --- | --- | --- | --- | --- | --- | --- | --- | --- | --- | --- |
| **#** | **Articles** | **YOP** | **Pt n°** | | **N° cases** | **Age at diagnosis** [*years*] | **Relapse/recurrance of disease** | **Site of relapse** | **Therapy at relapse** | **Time to relapse** [*years*] | **Secondary malignancies** | **Observations during follow-up** | **Last follow-up** [*years*] | **Outcome** | |
| **1** | Tamimi HK et al. | 1984 | **1** | | 1 | 15 |  |  |  |  |  |  | 0,6 | alive | |
| **2** | Pounder DJ et al. | 1985 | **2** | | 1 | 15 | contralateral metachronous JGCT |  | contralateral oophorectomy | 7 |  |  | 7 | alive | |
| **3** | Colombo N et al. | 1986 | **3** | | 1 | 7 | progression of disease | NS |  |  |  |  | 0,6 | DOD | |
| **4** | Vaz RM et al. | 1986 | **4** | | 1 | 8,5 |  |  |  |  |  |  | 0,2 | alive | |
| **5** | Schwartz HS et al. | 1987 | **5** | | 1 | 17 |  |  |  |  |  |  | 2 | alive | |
| **6** | Vassal G et al. | 1988 | **6** | | 15 | 1,8 |  |  |  |  |  |  | 9 | alive | |
|  |  |  | **7** | |  | 2 |  |  |  |  |  | HRT for pubertal progression | 14 | alive | |
|  |  |  | **8** | |  | 3,4 |  |  |  |  |  |  | 3 | alive | |
|  |  |  | **9** | |  | 4 |  |  |  |  |  |  | 2 | alive | |
|  |  |  | **10** | |  | 5,4 |  |  |  |  |  |  | 5 | alive | |
|  |  |  | **11** | |  | 6 |  |  |  |  |  |  | 8 | alive | |
|  |  |  | **12** | |  | 7,7 |  |  |  |  |  |  | 5 | alive | |
|  |  |  | **13** | |  | 9,3 | relapse | abdominal mass; bone and bone marrow metastases |  | 0.8 |  |  | NS | DOD | |
|  |  |  | **14** | |  | 10 |  |  |  |  |  |  | 2 | alive | |
|  |  |  | **15** | |  | 10,8 | relapse | abdominal mass; pathological lomboaortic and inguinal lymph nodes |  | 0.9 |  |  | NS | DOD | |
|  |  |  | **16** | |  | 11,5 |  |  |  |  |  |  | 18 | alive | |
|  |  |  | **17** | |  | 12,3 |  |  |  |  |  |  | 3 | alive | |
|  |  |  | **18** | |  | 13,9 |  |  |  |  |  |  | 3 | alive | |
|  |  |  | **19** | |  | 15,4 | relapse | abdominal mass; cervical, mediastinal and inguinal lymph nodes; lung lymphangitis |  | 0.5 |  |  | NS | DOD | |
|  |  |  | **20** | |  | 15,6 | relapse | abdominal mass; pathological lymph nodes and bone metastases | doxorubicin, cyclophosphamide, cisplatin, teniposide, vindesine, followed by ifosfamide, vincristine, dactinomycin and high-dose melphalan and aHSCT | 1.6 |  |  | NS | DOD | |
| **7** | Velasco-Oses A et al. | 1988 | **21** | | 1 | 6 |  |  |  |  |  |  | 7 | alive | |
| **8** | Asirvatham R et al. | 1991 | **22** | | 1 | 4 |  |  |  |  | low-grade chondrosarcoma of the fibula |  | 8 | alive | |
| **9** | Le Gall C et al. | 1991 | **23** | | 1 | 12 |  |  |  |  |  |  | NS | alive | |
| **10** | Tanaka Y et al. | 1992 | **24** | | 1 | 15 |  |  |  |  |  |  | 4 | alive | |
| **11** | Powell JL et al. | 1993 | **25** | | 1 | 13 |  |  |  |  |  |  | 7 | alive | |
| **12** | Wessalowski R et al. | 1995 | **26** | | 1 | 3 | relapse | liver and omental metastases | surgical resection, hyperthermic treatment, 6 cycles of PEI, followed by abdomen RT | 0.8 |  |  | 1,8 | alive | |
| **13** | Kukuvitis A et al. | 1995 | **27** | | 1 | 0,6 |  |  |  |  |  | CPP | 6,8 | alive | |
| **14** | Silverman LA et al. | 1996 | **28** | | 2 | 2 |  |  |  |  |  |  | NS | alive | |
|  |  |  | **29** | |  | 7,6 |  |  |  |  |  |  | 1,3 | alive | |
| **15** | Powell JL et al. | 1996 | **30** | | 2 | 13 |  |  |  |  |  |  | 3,2 | alive | |
|  |  |  | **31** | |  | 17 |  |  |  |  |  |  | 1 | alive | |
| **16** | Bouffet E et al. | 1997 | **32** | | 3 | 1,3 |  |  |  |  |  |  | 11 | alive | |
|  |  |  | **33** | |  | 1,3 |  |  |  |  |  |  | 7 | alive | |
|  |  |  | **34** | |  | 0,4 |  |  |  |  |  |  | 7 | alive | |
| **17** | Feilberg Jørgensen N et al. | 1998 | **35** | | 1 | 1,5 |  |  |  |  |  | CPP | NS | alive | |
| **18** | Gell JS et al. | 1998 | **36** | | 1 | 13 |  |  |  |  |  |  | 1 | alive | |
| **19** | Daubenton JD et al. | 2000 | **37** | | 1 | 11 | relapse | NS |  | 0.5 |  |  | 0,8 | DOD | |
| **20** | Powell JL et al. | 2001 | **38** | | 1 | 17 | relapse | liver and perisplenic area | radical surgery, followed by 6 cycles of bleomycin and taxol | 1 |  |  | 3 | alive | |
| **21** | Erdreich-Epstein A et al. | 2002 | **39** | | 1 | 10,5 |  |  |  |  |  |  | NS | NS | |
| **22** | Chan LF et al. | 2004 | **40** | | 1 | 3,9 |  |  |  |  |  |  | NS | alive | |
| **23** | Tenorio Romojaro V et al. | 2004 | **41** | | 1 | 1,3 |  |  |  |  |  |  | 0,3 | alive | |
| **24** | Koksal Y et al. | 2004 | **42** | | 1 | 11 |  |  |  |  |  |  | 1 | alive | |
| **25** | Kdous M et al. | 2004 | **43** | | 1 | 6 |  |  |  |  |  |  | 14 | alive | |
| **26** | Till H et al. | 2005 | **44** | | 1 | 6 |  |  |  |  |  |  | 3 | alive | |
| **27** | Larizza D et al. | 2006 | **45** | | 1 | 16 |  |  |  |  |  |  | 0,7 | alive | |
| **28** | Kdous M et al. | 2006 | **46** | | 1 | 6 |  |  |  |  |  |  | 14 | alive | |
| **29** | Guo H et al. | 2006 | **47** | | 1 | 8 | relapse | contralateral ovary | contralateral salpingo-oophorectomy, lymphadenectomy and omentectomy, followed by 4 cycles of PEB | 0.25 |  |  | 8 | alive | |
| **30** | Leyva-Carmona M et al. | 2009 | **48** | | 2 | 0,8 | relapse | left ovarian bed | surgery | 8 |  |  | 11 | alive | |
|  |  |  | **49** | |  | 0 |  |  |  |  |  |  | 3 | alive | |
| **31** | Pascual J et al. | 2009 | **50** | | 1 | 0,2 |  |  |  |  |  |  | NS | alive | |
| **32** | Capito C et al. | 2009 | **51** | | 1 | 0 | ipsilateral recurrence, contralateral metachronous JGCT |  | salpingo-oophorectomy | 0.12 |  |  | 2 | alive | |
| **33** | Hashemipour M et al. | 2010 | **52** | | 1 | 6 |  |  |  |  |  |  | NS | alive | |
| **34** | Wang Y et al. | 2011 | **53** | | 3 | 0,7 |  |  |  |  |  |  | NS | alive | |
|  |  |  | **54** | |  | 3 |  |  |  |  |  |  | 13 | alive | |
|  |  |  | **55** | |  | 4 |  |  |  |  |  |  | 8 | alive | |
| **35** | Paternoster M et al. | 2011 | **56** | | 1 | 0,8 |  |  |  |  |  |  | NS | DOD | |
| **36** | Calcaterra V et al. | 2013 | **57** | | 1 | 8,4 |  |  |  |  |  |  | 0,58 | alive | |
| **37** | Haroon NN et al. | 2013 | **58** | | 1 | 7 |  |  |  |  |  |  | 3,5 | alive | |
| **38** | Bedir R et al. | 2014 | **59** | | 1 | 10 |  |  |  |  |  |  | NS | alive | |
| **39** | Dhivyalakshmi J et al. | 2014 | **60** | | 1 | 0,7 |  |  |  |  |  |  | NS | alive | |
| **40** | Benesch M et al. | 2015 | **61** | | 1 | 4,5 | 2 relapses | 1) paraortic lymph nodes; 2) paratracheal lymph nodes | 1st relapse) surgery, local radiotherapy and 3 courses PEB, high-dose chemotherapy with carboplatin and etoposide followed by autologous stem cell support; 2nd relapse) surgical removal and mediastinal irradiation, chemotherapy (paclitaxel, bevacizumab, thalidomide and pegylated interferon) | 1st relapse) 1; 2nd relapse) 0.6 |  |  | 8 | alive | |
| **41** | Lamas-Pinheiro R et al. | 2016 | **62** | | 1 | 3 |  |  |  |  |  |  | 3 | alive | |
| **42** | Sampagar AA et al. | 2016 | **63** | | 1 | 2,5 |  |  |  |  |  | incidental findings of enchondromas | NS | alive | |
| **43** | Lacourt P et al. | 2017 | **64** | | 1 | 0,8 |  |  |  |  |  | non-specific transient thelarche | 4 | alive | |
| **44** | Drucker NA et al. | 2017 | **65** | | 1 | 0 |  |  |  |  |  |  | 2,5 | alive | |
| **45** | Wu H et al. | 2017 | **66** | | 8 | 1,4 |  |  |  |  | liposarcoma |  | 3,1 | death due to second malignancy | |
|  |  |  | **67** | |  | 2,6 |  |  |  |  |  |  | 14,3 | alive | |
|  |  |  | **68** | |  | 3,3 |  |  |  |  |  |  | 14,9 | alive | |
|  |  |  | **69** | |  | 12,8 |  |  |  |  |  |  | 9,4 | alive | |
|  |  |  | **70** | |  | 0,9 |  |  |  |  |  |  | 1,5 | alive | |
|  |  |  | **71** | |  | 14,3 | relapse | NS |  | 0.5 |  |  | 2,1 | DOD | |
|  |  |  | **72** | |  | 0,7 |  |  |  |  |  |  | 1,3 | alive | |
|  |  |  | **73** | |  | 9,2 | relapse |  | debulking surgery |  |  | liver hemangioma | 9,3 | alive | |
| **46** | Mohapatra A et al. | 2019 | **74** | | 1 | 5 |  |  |  |  |  |  | 3 | alive | |
| **47** | Andreetta M et al. | 2020 | **75** | | 1 | 5,8 |  |  |  |  | metachronous benign mucinous cystadenoma |  | 3 | alive | |
| **48** | Okawa ER et al. | 2020 | **76** | | 1 | 15,8 |  |  |  |  |  |  | 0,25 | alive | |
| **49** | Kwiatkowska A et al. | 2020 | **77** | | 1 | 17 | relapse | ipsilateral ovary | unilateral oophorectomy | 0.75 |  |  | NS | alive | |
| **50** | Jalaeefar A et al. | 2020 | **78** | | 1 | 17 |  |  |  |  |  |  | NS | alive | |
| **51** | Ben David Y et al. | 2021 | **79** | | 1 | 0 |  |  |  |  |  |  | NS | alive | |
| **52** | Viet LA et al. | 2021 | **80** | | 1 | 4 |  |  |  |  | contralateral ovarian serous cystadenoma |  | 1 | alive | |
| **53** | Barakizou H et al. | 2021 | **81** | | 1 | 2,2 |  |  |  |  |  |  | 6 | alive | |
| **54** | De Sanctis V et al. | 2021 | **82** | | 1 | 0,8 |  |  |  |  |  |  | 1 | alive | |
| **55** | Hovsepyan S et al. | 2021 | **83** | | 2 | 16 |  |  |  |  |  |  | NS | alive | |
|  |  |  | **84** | |  | 15 | relapse | ipsilateral ovary | unilateral adnexectomy and omentectomy, followed by 4 cycles of PEI |  |  |  | NS | alive | |
| **56** | Gaikwad et al. | 2022 | **85** | | 1 | 5.4 |  |  |  |  |  | CPP | 1,5 | alive | |
| **57** | Qutub LM et al. | 2022 | **86** | | 1 | 6 |  |  |  |  |  |  | NS | alive | |
| **58** | Kim RC et al. | 2022 | **87** | | 1 | 4 |  |  |  |  |  |  | 2 | alive | |
| **59** | Khatun F et al. | 2022 | **88** | | 1 | 4 |  |  |  |  |  |  | NS | NS | |
| **60** | Littrell LA et al. | 2023 | **89** | | 1 | 15 |  |  |  |  |  |  | 0,75 | alive | |
| **61** | Zhang J et al. | 2023 | **90** | | 1 | 4 |  |  |  |  |  |  | 2 | alive | |
| **62** | Park H et al. | 2023 | **91** | | 1 | 12 |  |  |  |  |  |  | NS | alive | |
| **63** | Devins KM et al. | 2024 | **92** | | 1 | 10 |  |  |  |  |  |  | NS | NS | |
| **64** | Shero N et al. | 2024 | **93** | | 1 | 14 | 3 relapses | ipsilateral ovary | Surgery: radical cytoreductive surgery. For tumor rupture, second cytoreductive surgery, contralateral salpingo-oophorectomy, small bowel resection, peritonectomy and hyperthermic intraperitoneal chemotherapy with cisplatin. Chemotherapy: VAC/VI; 6 cycles of VAC/. At 3rd relapse, gemcitabine and nab-paclitaxel for rhabdomyosarcomatous relapse. | 3 |  |  | 4 | DOD | |
| **65** | Amirkashani D et al. | 2024 | **94** | | 1 | 6 |  |  |  |  |  |  | 0,2 | alive | |

[YOP – year of publication; NS – not specified; DOD – died of disease; CPP – central precocious puberty; aHSCT – autologous Hematopoietic Stem Cell Transplantation; HRT – hormone replacement therapy; VAC – vincristine, dactinomycin and cyclophosphamide; VI – vincristine and irinotecan]
